# *Microcystis aeruginosa msoT1/msoA1* Locus Displays Features of a Type I Toxin–Antitoxin System

**DOI:** 10.3390/toxins17080360

**Published:** 2025-07-22

**Authors:** Matija Ruparčič, Marko Dolinar

**Affiliations:** Department of Chemistry and Biochemistry, Faculty of Chemistry and Chemical Technology, University of Ljubljana, Večna pot 113, 1000 Ljubljana, Slovenia; matija.ruparcic@ki.si

**Keywords:** type I toxin–antitoxin systems, cyanobacteria, *Microcystis aeruginosa*, bacterial toxins, novel toxin–antitoxin systems

## Abstract

Type I toxin–antitoxin (TA) systems consist of a protein toxin that exerts a cytostatic or cytotoxic effect and an antisense RNA antitoxin that prevents translation of the toxin. Although well studied, type I TA systems have so far only been discovered in bacteria from the phyla Proteobacteria, Firmicutes, and Tenericutes. We hypothesized that type I systems could also be present in Cyanobacteria. Through bioinformatic analysis of the *Microcystis aeruginosa* PCC 7806SL genome, we discovered ten putative type I TA loci and characterized six of them experimentally. Two of the six putative type I toxins, BH695_0320 and MsoT1 (BH695_4017), were observed to negatively affect *Escherichia coli* cell growth, with MsoT1 exerting a phenotype similar to SrnB, a known type I toxin. We focused on the MsoT1/MsoA1 TA system and confirmed the expression of MsoT1 and MsoA1 in our assay. Additionally, we found that MsoA1 delays the toxic effects of MsoT1, indicating its role as a cognate antitoxin of MsoT1. Our results suggest that MsoT1/MsoA1 represents a novel candidate type I TA system, the first to be discovered in the Cyanobacteria phylum.

## 1. Introduction

Toxin–antitoxin (TA) systems are two-gene modules that encode a stable toxin, which stops cell growth or causes cell death, and a labile antitoxin, which inhibits the expression or activity of the toxin [1]. They were originally discovered on plasmids [2,3], but have since been identified on various bacterial and archaeal chromosomes [4,5,6]. Plasmid-encoded TA systems promote the maintenance of their replicon by killing cells that have not inherited a plasmid copy, a process known as “post-segregational killing” (PSK) [2,7,8,9]. Chromosomal TA systems have been linked to several cellular processes, including abortive infection [10,11], neutralization of PSK by plasmid-encoded TAs [12], and bacterial persistence [13]. TA systems are currently classified into eight different types (I-VIII), depending on the nature and mechanism of action of the antitoxin. In type II, IV, V, VI, and VII systems, the antitoxin is a protein, whereas in type I, III, and VIII systems, it is a non-coding RNA. In almost all currently known TA systems, the toxin is a protein, with the exception of type VIII, where it is a non-coding RNA [14].

In type I systems (reviewed in [15]), the antitoxin is an antisense RNA that is transcribed from its own promoter separately from its cognate toxin. It is encoded on the opposite strand, where it can either overlap with the toxin gene (*cis*-encoded) or be encoded in proximity of the toxin gene without overlap (*trans*-encoded). The main feature of type I antitoxins are their complementarity regions with the cognate toxin mRNA, which enable them to form base pairs with the toxin transcript. The formation of the RNA-mRNA duplex prevents translation of the toxin in two ways. Most of the currently known type I toxins act by preventing the ribosome from accessing the ribosome binding site (RBS), thus disabling translation of the toxin. In the case of the SymE/SymR, Ibs/Sib, and Hok/Sok systems, the antitoxin forms base pairs with the RBS sequence of either the toxin [16,17] or the leader peptide gene [8], thus blocking ribosome binding. Similarly, the antitoxins of the TisB/Ist-R1 and BsrG/SR4 systems also prevent ribosome binding, albeit in an indirect manner. In the first case, the Ist-R1 antitoxin binds to a ribosome standby site (RSS) upstream of the RBS [18], whereas in the second case, SR4 binds to the 3′-untranslated region (3′-UTR) of the toxin mRNA, leading to conformational changes in the structure of the mRNA, resulting in the RBS being sequestered in a hairpin loop where it is inaccessible to the ribosome [19]. Other type I antitoxins, such as those from the TxpA/RatA and YonT/SR6 systems, do not block ribosome binding. Instead, the RNA-mRNA duplex formed is recognized by the enzyme RNase III, which degrades the toxin mRNA [20,21].

Type I toxins are generally short hydrophobic peptides containing an α-helical transmembrane domain [22,23]. They exert their cytotoxic effects in two ways: some disrupt membrane integrity by forming pores [24,25], while others inhibit replication and transcription by inducing nucleoid condensation [26,27]. The two exceptions are SymE and RalR, which are cytosolic proteins that cleave RNA and DNA, respectively [28,29].

Type I TA systems, together with type II, are the best described TA systems, both of which have a large number of annotated systems in their respective databases (T1TAdb for type I [30] and TADB 3.0 for type II [31]). Interestingly, type I systems appear to have a significantly shorter phylogenetic range compared to type II. While type II TA systems are found in numerous bacterial phyla, type I TA systems have only been identified in Proteobacteria, Firmicutes, and Tenericutes, with many of them occurring in less than 5% of species within these phyla. However, this apparent narrowness of type I TA systems is thought to be due to a bias in the methods used to discover type I loci, which rely on searching for loci that have sequence-based features similar to already known type I loci. In addition, type I systems have historically been difficult to predict in silico due to the small size of the toxins (less than 60 amino acids) and the lack of a tool to predict their antisense RNA antitoxin counterparts [15,32].

We hypothesized that bacteria from other phyla might also possess type I TA systems that are not closely related to the currently known systems. To test this hypothesis, we chose the cyanobacterium *Microcystis aeruginosa* PCC 7806SL, of which our group had already successfully characterized a type II TA system [33]. In addition, research on the model cyanobacterium *Synechocystis* sp. PCC 6803 has shown that many genes of this cyanobacterium are regulated by antisense RNAs [34]. If this is true for all cyanobacteria, it would further support the possibility of the presence of type I TA systems in *M. aeruginosa*.

The aim of this study was to identify new putative type I TA systems in *Microcystis aeruginosa* PCC 7806SL. We performed a bioinformatic analysis in which we searched the genome of *M. aeruginosa* PCC 7806SL for novel putative type I TA loci using a set of search parameters based on the characteristics of known type I TA systems. We identified ten putative type I TA loci and characterized six of them experimentally in *Escherichia coli*, namely BH695_0311/MaTIA_0311, BH695_0320/MaTIA_0320, BH695_3336/MaTIA_3336, BH695_4017/MaTIA_4017, BH695_4989/MaTIA_4989, and BH695_5020/MaTIA_5020 (the nomenclature is explained in the Materials and Methods section).

Our results show that the expression of BH695_0320 and BH695_4017 has a negative effect on cell growth, while the expression of BH695_0311, BH695_3336, BH695_4989, and BH695_5020 has no effect. We continued our research focusing on the BH695_4017/MaTIA_4017 pair, which we renamed to MsoT1/MsoA1. We show that the toxic effect of MsoT1 expression is delayed when MsoA1 is constitutively expressed in the cell. Furthermore, we show that two reporter proteins, β-lactamase and monomeric red fluorescent protein (mRFP), are constitutively expressed when their coding sequences are placed under the control of the predicted MsoA1 antitoxin promoters, demonstrating their biological efficiency.

## 2. Results

### 2.1. Predicted Type I TA Loci in the Genome of Microcystis aeruginosa PCC 7806SL

**Figure 1:**
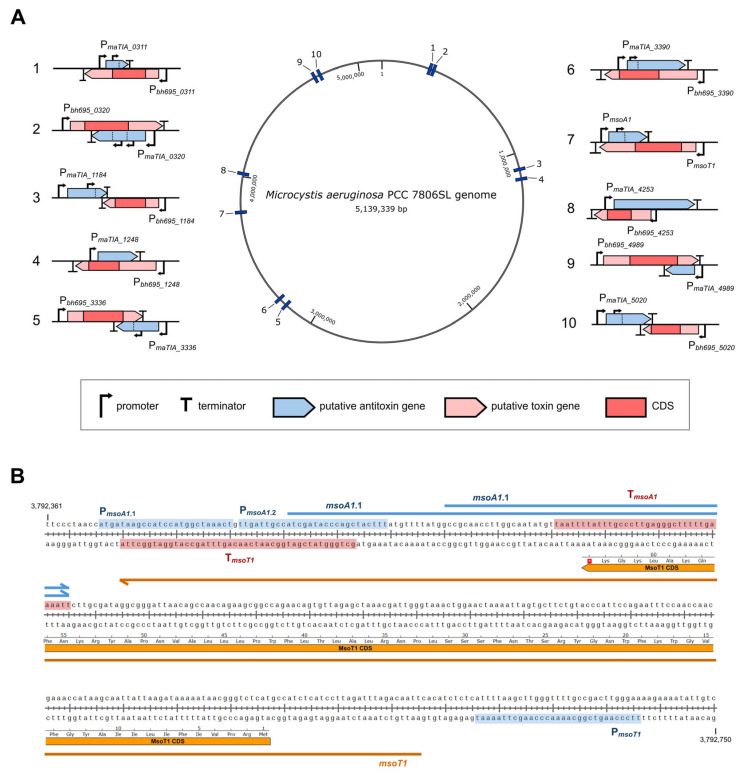
(**A**) Genomic distribution of the ten putative type I TA systems from *M. aeruginosa* PCC 7806SL with schematics showcasing the organization of each locus. (**B**) Nucleotide sequence of the *msoT1/msoA1* locus. The sequences of predicted promoters and terminators are highlighted in blue and red, respectively. The predicted MsoT1 and MsoA1 genes are shown as orange and blue arrows, respectively. The MsoT1 CDS with the translated amino acid sequence is presented as an orange ribbon and was created with SnapGene (v8.1.1).

Using our de novo bioinformatic analysis, we hypothesized the presence of ten regions in the genome of *M. aeruginosa* PCC 7806SL which we suspected to contain a type I TA locus (Table 1 and Table 2). The putative type I toxins ranged in length from 39 (BH695_3390) to 81 (BH695_4989) amino acids. Most of them were predicted to have one transmembrane helix, with the exception of BH695_4017 and BH695_4989, which were predicted to have two. The length of the cognate putative type I antitoxins ranged from 43 (MaTIA_0311_1 and MaTIA_1184_1) to 252 (MaTIA_4253) nucleotides, seven of which had multiple predicted promoters, indicating the possibility of different isoforms. Six putative antitoxins overlapped with the CDS and the 3′-untranslated region (3′-UTR) of the toxin mRNA, three overlapped with the CDS and the 5′-UTR, and one overlapped only with the 3′-UTR (Figure 1).

For experimental characterization, we selected BH695_0311/MaTIA_0311, BH695_0320/MaTIA_0320, BH695_4017/MaTIA_4017, BH695_4989/MaTIA_4989, and BH695_5020/MaTIA_5020, as they were considered the most promising due to their predicted promoters and terminators. In addition, we selected BH695_3336/MaTIA_3336 because the adjacent gene on the opposite strand, BH695_3337, encodes the ATP-binding subunit ClpC of the Clp protease, which is known to cleave type II antitoxins [35,36].

### 2.2. Overexpression of BH695_0320 and BH695_4017 Negatively Affects Escherichia coli Cell Growth

**Figure 2:**
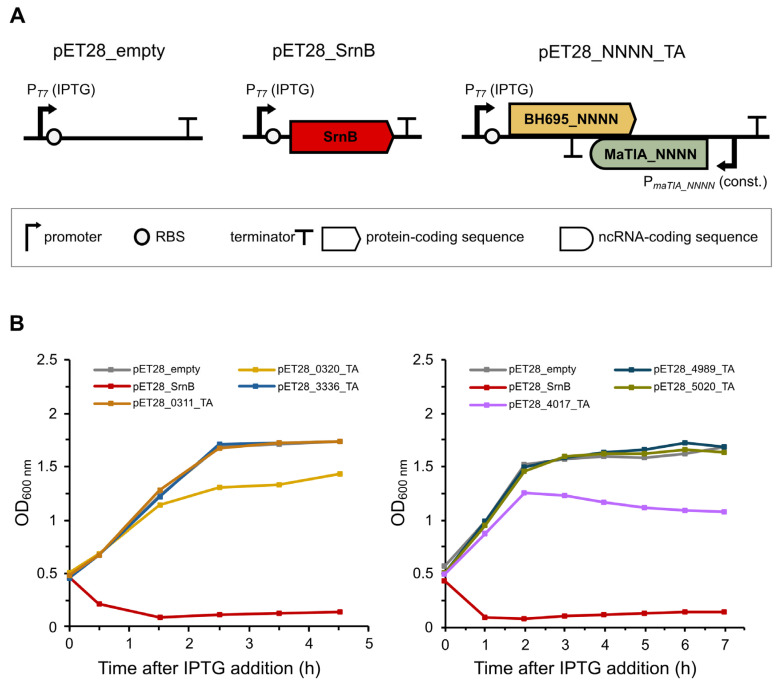
Expression of six putative type I toxins of *M. aeruginosa* PCC 7806SL and their effect on *E. coli* BL21(DE3) pLysS cell growth. (**A**) Schematic representation of the pET28b(+) plasmid constructs used. The plasmid without an insert was used as a negative control. For the positive control, the sequence encoding the SrnB type I toxin from *E. coli* was inserted under the control of the IPTG-inducible T7 promoter. For the test samples, the entire *M. aeruginosa* putative type I TA locus was inserted, with the putative type I toxin under the control of the IPTG-inducible T7 promoter and the putative type I antitoxin under the control of its predicted constitutive promoters. (**B**) Growth curves of *E. coli* BL21(DE3) pLysS cells following the induction of protein expression with IPTG.

We assessed the activity of the six selected putative type I toxins by expressing them in *Escherichia coli* BL21(DE3) pLysS cells from the IPTG-inducible T7 promoter and observing their effect on cell growth. For the negative control, cells transformed with the pET28_empty plasmid were used, while cells expressing the SrnB type I toxin were used for the positive control (Figure 2A). To avoid prolonged pauses in cell culturing due to too many cultures being taken at once, the assay was performed twice: first for BH695_0311, BH695_0320, and BH695_3336, and second for BH695_4017, BH695_4989, and BH695_5020 (Figure 2B). Cells were grown in liquid M9+CK medium.

After addition of IPTG (at an OD_600_ of approximately 0.5), cells harboring the pET28_empty plasmid continued to grow normally as expected (Figure 2B, grey lines), as no toxic protein was expressed from the T7 promoter. In contrast, a rapid decrease in OD_600_ was observed after induction in cells containing the pET28_SrnB plasmid (Figure 2B, red lines), consistent with the bacteriolytic effect of SrnB [37]. In addition, filamentous clumps were observed in the liquid medium of the pET28_SrnB-transformed cells shortly after induction, further indicating phenotypic effects of cell lysis [38]. Similar to the negative control, cells containing the plasmids pET28_0311_TA, pET28_3336_TA, pET28_4989_TA, and pET28_5020_TA grew normally after induction with IPTG (Figure 2B, orange, blue, teal, and green lines, respectively), indicating that in our experiment BH695_0311, BH695_3336, BH695_4989, and BH695_5020 are not toxic to *E. coli* cells. In contrast, the growth rates of cells containing the plasmids pET28_0320_TA and pET28_4017_TA were negatively affected after induction with IPTG. Expression of BH695_0320 resulted in impaired cell growth 150 min after induction, indicating a mild bacteriostatic effect (Figure 2B, yellow line), while expression of BH695_4017 resulted in a reduced growth rate 120 min after induction, at which time filamentous clumps also appeared in the culture, possibly indicating a mild bacteriolytic effect (Figure 2B, purple line).

For the remaining experiments, we decided to focus on the putative type I TA system BH695_4017/MaTIA_4017, as the expression of BH695_4017 resulted in a phenotype most similar to that of the positive control. Furthermore, the putative toxin was renamed MsoT1 (*Microcystis* short ORF encoding a toxin 1), and its cognate antitoxin was renamed MsoA1 (MsoT1 antitoxin 1).

### 2.3. Constitutive Expression of MsoA1 Delays Toxic Effect of MsoT1 Expression in Escherichia coli

**Figure 3:**
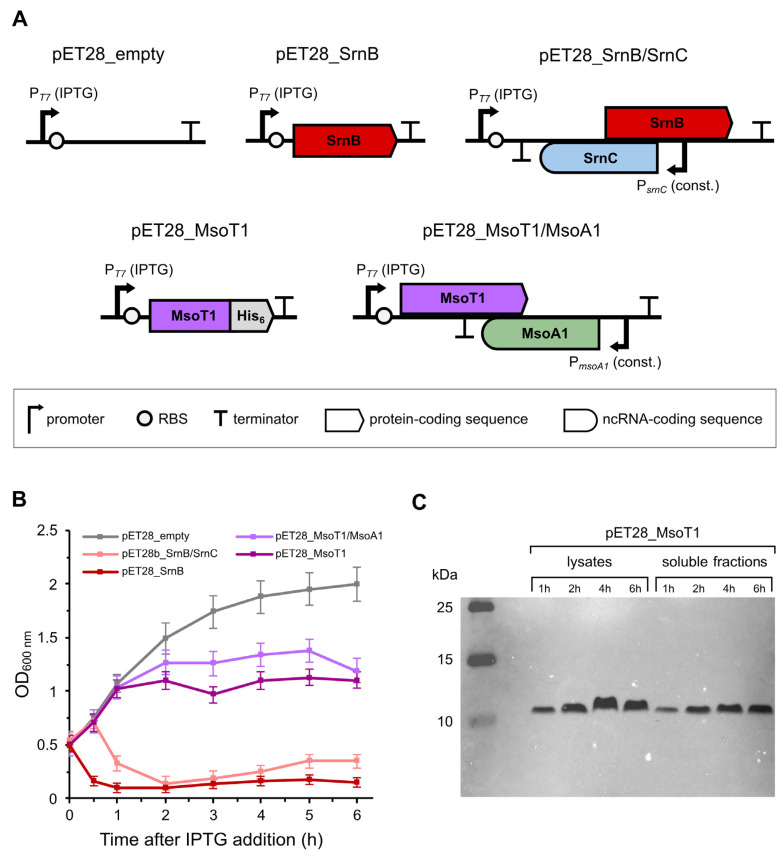
Expression of the SrnB type I toxin from *E. coli* in the presence or absence of its cognate SrnC antitoxin and of the MsoT1 (BH695_4017) putative type I toxin from *M. aeruginosa* in the presence and absence of its cognate MsoA1 antitoxin in *E. coli* BL21(DE3) pLysS cells. (**A**) Schematic representation of the pET28b(+) plasmid constructs used in the cell toxicity assay. Cells transformed with the empty plasmid were used as a negative control. For the first positive control, the sequence encoding the SrnB type I toxin from *E. coli* was inserted under the control of the IPTG-inducible T7 promoter. For the second positive control, the entire *srnB/srnC* type I TA locus from *E. coli* was inserted, with SrnB under the control of the IPTG-inducible T7 promoter and its cognate SrnC antitoxin under the control of its endogenous constitutive promoter. For the first test sample, the coding sequence of the MsoT1 (BH695_4017) putative type I toxin from *M. aeruginosa* was inserted under the control of the IPTG-inducible T7 promoter. The construct also introduced a C-terminal His-tag, which enabled immunodetection of the putative toxin. For the second test sample, the entire MsoT1/MsoA1 putative type I TA locus of *M. aeruginosa* was inserted, with the putative MsoT1 toxin under the control of the IPTG-inducible T7 promoter and its cognate MsoA1 antitoxin under the control of its predicted constitutive promoter. (**B**) Growth curves of *E. coli* BL21(DE3) pLysS cells following the induction of toxin expression with IPTG. Values represent mean ± SE of 3 biological replicates. (**C**) Immunodetection of the His-tagged MsoT1 with HRP-conjugated anti-His antibodies. Aliquots of cells containing the pET28_MsoT1 plasmid were harvested 1, 2, 4, and 6 h after induction with IPTG to prepare cell lysates and soluble fractions.

We assessed the antitoxic effect of the putative type I antitoxin MsoA1 by comparing the growth curves of *Escherichia coli* BL21(DE3) pLysS cells expressing the MsoT1 toxin from the IPTG-inducible T7 promoter in the presence and absence of MsoA1 constitutively expressed from its predicted endogenous promoter. Cells transformed with the plasmids pET28_empty and pET28_SrnB again served as the negative and the positive control, respectively. An additional positive control was used to compare how a known type I antitoxin affects the toxic effects of its cognate type I toxin. For this additional positive control, we used cells expressing the SrnB toxin from the IPTG-inducible T7 promoter and its cognate SrnC antitoxin from its endogenous constitutive P*_srnC_* promoter (Figure 3A). This time, cells were grown in the richer liquid M9++CK medium to allow bacteria to reach stationary phase later, resulting in a more pronounced difference between the growth curves of the negative control and the test samples due to the late and relatively mild toxic effects of the cyanobacterial MsoT1 on *E. coli* cells. The assay was performed with three biological replicates.

After the addition of IPTG, the negative and the first positive control showed results comparable to the first assay, i.e., normal growth of cells transformed with the pET28_empty plasmid (Figure 3B, white line) and a rapid decrease in OD_600_ in cells carrying the pET28_SrnB plasmid (Figure 3B, red line), respectively. In cells transformed with the pET28_SrnB/SrnC plasmid (second positive control), induction with IPTG also led to a rapid decrease in OD_600_ (Figure 3B, pink line). Compared to the first positive control, the decrease occurred with a delay of about 30 to 60 min, confirming the antitoxic effect of SrnC, which can only neutralize the highly expressed toxin for a limited time. In the test samples containing MsoT1/MsoA1 constructs, cell growth slowed down 120 min after induction, similar to the first assay. After induction, a slight but significant difference was observed between the cell growth of the MsoT1 and MsoT1/MsoA1 constructs. In all three replicates, the growth rate of cells harboring the pET28_MsoT1/MsoA1 plasmid (Figure 3B, purple line) slowed down with a slight delay compared to cells transformed with pET28_MsoT1 (Figure 3B, dark purple line). These results, when compared to those of the two positive controls, suggest that MsoA1 acts as a cognate antitoxin for MsoT1 by delaying its toxic effects, similar to the SrnB/SrnC type I TA system.

Moreover, after induction, filamentous clumps were again observed in the cultures of cells transformed with the plasmids pET28_SrnB, pET28_SrnB/SrnC, pET28_MsoT1, and pET28_MsoT1/MsoA1, possibly indicating cell lysis. To further verify this, an aliquot of each sample was stained with erythrosin B—a dye that specifically stains non-viable or membrane-damaged bacteria pink [39]. When the samples were viewed under a bright-field microscope, an abundance of pink-colored clumps was observed in the cultures of cells transformed with the plasmids pET28_SrnB/SrnC, pET28_SrnB, pET28_MsoT1/MsoA1, and pET28_MsoT1, confirming the bacteriolytic effect of the toxins SrnB and MsoT1. A significantly lower number of erythrosin B-stained clumps was also observed in the cultures of cells transformed with the pET28_empty plasmid, which may be attributed to cell death during the stationary phase of bacterial growth (Supplementary Figure S7).

We confirmed our findings on the negative effects of MsoT1 expression on *E. coli* cells by confirming the expression of MsoT1 at the protein level using *western* blot and immunodetection. Since the construct introduced a His-tag on the C-terminus of the protein, we were able to specifically detect MsoT1 with anti-His antibodies (Figure 3A). When the blotted membrane was analyzed, a single band corresponding to the His-tagged MsoT1 toxin was observed in all lanes of both the lysates and the soluble fractions (Figure 3C). In addition, the intensity of the bands increased with time after induction, indicating an increase in the amount of MsoT1 with time after induction. Comparison of the bands of the lysates and the soluble fractions showed a lower intensity in the latter, suggesting that some part of the protein could also be present in the insoluble fraction. Interestingly, the His-tagged MsoT1 migrated more slowly through the gel during SDS-PAGE as expected. While the bands were positioned just above the 10 kDa standard, the calculated molecular mass of the protein is approximately 8.8 kDa.

### 2.4. The Sequence Upstream of MsoA1 Contains Biologically Active Constitutive Promoters

**Figure 4:**
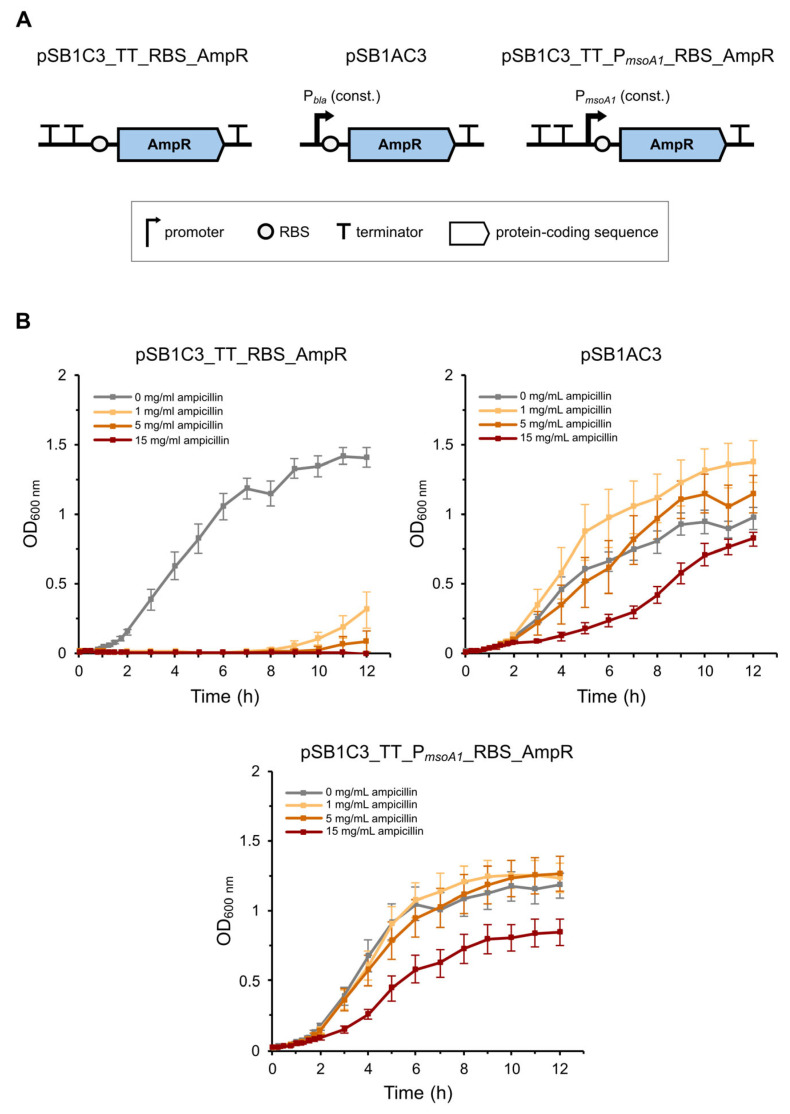
Growth of *E. coli* BL21(DE3) cells expressing β-lactamase from the P*_bla_* or P*_msoA1_* constitutive promoter in media with and without ampicillin. (**A**) Schematic representation of the plasmid constructs used. For the negative control, the RBS and the CDS of β-lactamase were inserted into the pSB1C3 plasmid without a dedicated promoter. For the positive control, the pSB1AC3 plasmid was used, in which β-lactamase is under the control of its P*_bla_* endogenous constitutive promoter. For the test sample, the RBS and the CDS of β-lactamase were inserted into the pSB1C3 plasmid under the control of the P*_msoA1_* predicted constitutive promoter(s). In the case of pSB1C3_TT_RBS_AmpR and pSB1C3_TT_P*_msoA1_*_RBS_AmpR, two bacterial terminators were also inserted just upstream of the RBS-CDS to prevent transcription from potential cryptic promoter(s) on the plasmid backbone. (**B**) The expression of β-lactamase was assessed by monitoring *E. coli* BL21(DE3) cell growth in media containing 0, 1, 5, or 15 mg/mL of ampicillin. Values represent mean ± SE of 12 sets of measurements (6 biological replicates in duplicates) for pSB1C3_TT_RBS_AmpR and pSB1C3_TT_*P_msoA1_*_RBS_AmpR and 8 sets of measurements (3 biological replicates, once in duplicates and twice in triplicates) for pSB1AC3.

**Figure 5:**
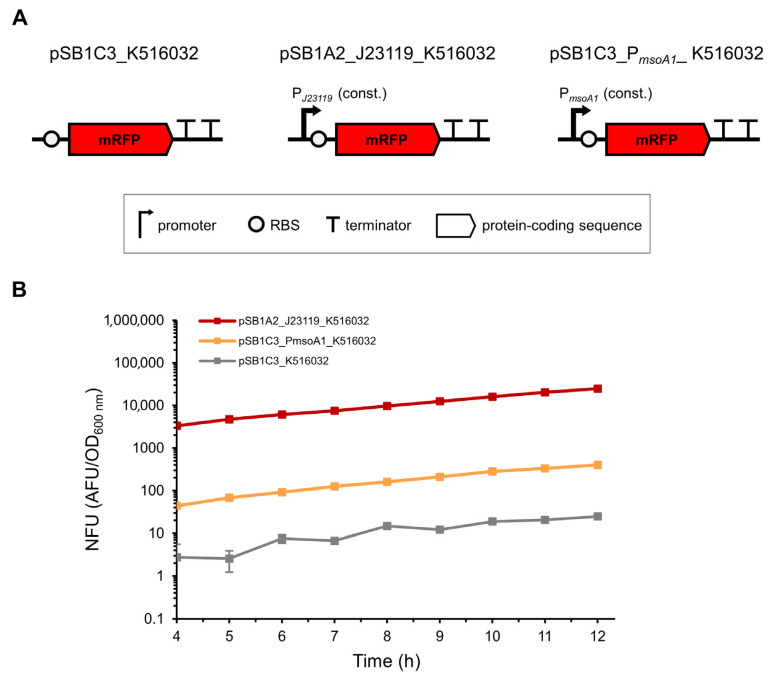
mRFP expression from the P*_J23119_* and P*_msoA1_* constitutive promoters in *E. coli* BL21(DE3) cells. (**A**) Schematic representation of the plasmid constructs used. For the negative control, the BBa_K516032 BioBrick containing a medium efficiency RBS and the coding sequence for mRFP was inserted into the pSB1C3 plasmid without a dedicated promoter. For the positive control, the BBa_K516032 BioBrick was inserted into the pSB1A2 plasmid just downstream of the BBa_J23119 BioBrick, which contains a strong constitutive promoter. For the test sample, the BBa_K516032 BioBrick was inserted into the pSB1C3 plasmid under the control of the P*_msoA1_* predicted constitutive promoters. (**B**) The expression of mRFP was assessed by measuring red fluorescence of *E. coli* BL21(DE3) cells. For normalization, the absolute fluorescence unit (AFU) at each time point was divided by the OD_600_ measured at the same time point, resulting in a normalized fluorescence unit (NFU). Values represent mean ± SE from 20 sets of measurements (4 biological replicates in quintuplicates).

In contrast to MsoT1, the expression of MsoA1 could not be confirmed by immunodetection as the putative type I antitoxin is a non-coding RNA. Therefore, we designed an experiment to indirectly confirm the expression of MsoA1 by assessing whether the predicted constitutive promoters P*_msoA1_* are biologically active and therefore enable the expression of two reporter proteins (β-lactamase or mRFP). For the positive controls, we used cells expressing the reporter proteins from known promoters, while cells lacking a promoter to express the reporter proteins were used as negative controls (Figure 4A and Figure 5A).

In the first antitoxin promoter activity assay, using β-lactamase as reporter protein, cells transformed with the plasmids pSB1C3_P*_msoA1_*_RBS_AmpR (test sample), pSB1C3_RBS_AmpR (negative control), and pSB1AC3 (positive control) were used. Interestingly, although the negative control cells lacked a promoter to express β-lactamase, they grew normally in all media, including those containing the antibiotic ampicillin (Supplementary Figure S8). This suggested the presence of a cryptic promoter in the plasmid. To circumvent this problem, plasmids pSB1C3_P*_msoA1_*_RBS_AmpR and pSB1C3_RBS_AmpR were redesigned by adding two *rnpB M1* terminators from *E. coli* to the beginning of the insert to prevent expression of β-lactamase from potential cryptic promoter(s) upstream of the AmpR coding region on the plasmid backbone. The initial assay was repeated with the newly constructed pSB1C3_TT_P*_msoA1_*_RBS_AmpR and pSB1C3_TT_RBS_AmpR plasmids. In addition, higher concentrations of ampicillin were used. This time, the cells of both controls grew as expected—the cells with the pSB1C3_TT_RBS_AmpR plasmid (negative control) showed normal cell growth only in the medium without ampicillin, while the cells transformed with pSB1AC3 (positive control) grew in all media, with the slowest growth observed in the medium with 15 mg/mL ampicillin. Similarly to the positive control, cell growth was also observed in cells carrying the pSB1C3_TT_P*_msoA1_*_RBS_AmpR plasmid in all media—but significantly slower in LBC with 15 mg/mL ampicillin—indicating constitutive expression of β-lactamase from the predicted antitoxin promoters. Surprisingly, some replicates of the negative control cells started to grow after 8 h of cultivation in media containing 1 or 5 mg/mL ampicillin (Figure 4B).

To further confirm the biological activity of the predicted P*_msoA1_* promoters, we performed the promoter activity assay with an additional reporter protein. Since the expression of β-lactamase could only be monitored indirectly by measuring cell growth in ampicillin-containing media, we chose mRFP as a second reporter protein because its expression could be monitored directly by measuring red fluorescence. Cells transformed with the plasmids pSB1C3_P*_msoA1_*_K516032 (test sample), pSB1C3_K516032 (negative control), and pSB1A2_J23119_K516032 (positive control) (Figure 5A) were used. To compensate for the differences in red fluorescence due to unequal bacterial growth, the absolute fluorescence units (AFU) were normalized by dividing them by the OD_600_ values. This resulted in the normalized fluorescence units (NFUs) being unusually high at low cell densities, which is why only the NFUs after 4 h of cultivation are shown (Figure 5B). When comparing the NFUs of all three samples, cells carrying the pSB1A2_J23119_K516032 plasmid (positive control) showed the highest emission of red light, which increased with time, indicating relatively high mRFP expression from the strong constitutive P*_J23119_* promoter (Figure 5B, red lines). Similarly, red light emission in cells containing the pSB1C3_P*_msoA1_*_K516032 plasmid (test sample) also increased with time, but the intensity was two orders of magnitude lower compared to the positive control, indicating a relatively weak expression of mRFP from the predicted P*_msoA1_* antitoxin promoter(s) (Figure 5B, orange lines). Interestingly, a temporal increase in red fluorescence was also observed in cells transformed with the pSB1C3_K516032 plasmid (negative control), but the intensity was three and one order of magnitude lower compared to the positive control and the test sample, respectively (Figure 5B, white lines), further supporting the biological activity of the predicted antitoxin promoters P*_msoA1_*.

### 2.5. Properties of the MsoT1/MsoA1 Toxin–Antitoxin Pair

The newly identified MsoT1 type I toxin comprises 63 amino acid residues and has a relatively high theoretical isoelectric point (pI) of 11.17. When analyzed with Alpha Fold 3 [40], it is predicted to fold into a structure consisting predominantly of α-helices (Supplementary Figure S9A). Interestingly, it is predicted to contain two membrane helices, which has not previously been reported for type I toxins. Moreover, the Alpha Fold 3 structure suggests that the second membrane helix may not penetrate the membrane bilayer but instead be embedded in it, similar to monotopic membrane proteins. Using the MsoT1 amino acid sequence as a BLASTp (v2.12.0) query, more than 100 hits were obtained. Most of them belong to predicted proteins from different *M. aeruginosa* strains, while the three most similar predicted proteins from different bacterial genera belong to *Chroococcus* sp. FPU101 (GenBank accession code: WP_206813397.1; 68% sequence identity), *Aphanothece hegewaldii* (GenBank accession code: WP_106455834.1; 64% sequence identity), and *Gloeotrichia echinulata* DEX184 (GenBank accession code: MCM0589651.1; 59% sequence identity), which all belong to the Cyanobacteria phylum. All four amino acid sequences are highly conserved among each other and show little to no similarity to other well-studied type I toxins (Supplementary Figure S9B). The cognate MsoA1 antitoxin is *cis*-encoded and is predicted to consist of two isoforms (56 and 83 nt long, respectively), that can bind to the 3′-end of the toxin coding region as well as to the 3′-UTR. Both isoforms are thought to form two hairpin loops (Supplementary Figure S9C).

## 3. Discussion

Type I toxin–antitoxin (TA) systems are one of the best-studied TA systems, having been discovered four decades ago [2,3]. Nevertheless, all currently known type I TA systems have only been identified in three bacterial phyla—Proteobacteria, Firmicutes, and Tenericutes—as it is difficult to predict these systems in bacterial genomes in silico [15,32]. In this work, we searched the genome of *Microcystis aeruginosa* PCC 7806SL, a cyanobacterium with an experimentally characterized type II TA system, for potential novel type I TA systems.

We performed a bioinformatic analysis in which we searched the list of predicted proteins of *M. aeruginosa* PCC 7806SL for proteins with features similar to already known type I toxins. This was done in a similar manner to the bioinformatic analysis of Fozo et al., who searched 774 bacterial genomes belonging to Proteobacteria and Firmicutes for novel type I TA systems [5]. Since cyanobacteria are not closely related to the bacteria studied by Fozo, we decided to make our filters less stringent to account for the possibility of greater divergence between the characteristics of known type I toxins and the potential toxins from cyanobacteria. Our filters differed from the filters used by Fozo as follows: (i) the upper limit for toxin size was increased from 70 to 90 amino acid residues; (ii) proteins with more than one predicted transmembrane segment were also considered; (iii) distances between neighboring genes (>400 nt upstream and >250 downstream set by Fozo) were not considered because recently discovered type I toxins such as RalR and YonT are encoded in proximity to neighboring genes [21,29]; and (iv) the C-terminal composition scoring was not used. As this is the first analysis of a cyanobacterial genome targeting type I TA systems, we searched for candidate type I TA systems in which the toxin is a transmembrane protein to avoid overgeneration of candidate type I systems that could burden the experimental characterization. Therefore, potential type I systems in which the toxin is a cytosolic protein, such as *symE/symR* and *ralR/ralA* from *Escherichia coli* [28,29], were intentionally omitted. For the prediction of cognate type I antitoxins, we chose a different approach than Fozo, as theirs again depended on the properties of previously known type I antitoxins, while we assumed that cyanobacterial antitoxins might differ from these. Therefore, we used four prediction tools—two for promoters and two for terminators—to predict the transcription start and termination sites of the putative type I antitoxins. However, a drawback of this approach is that it was developed only for the prediction of type I TA systems where the toxin and antitoxin genes overlap (the antitoxin is *cis*-encoded). For the prediction of type I TA systems where the toxin and antitoxin genes do not overlap (the antitoxin is *trans*-encoded), such as *dinQ/agrB* and *tisB/istR-1* from *Escherichia coli* [15], a different method would have to be developed.

Our bioinformatic search ended with a scored list of theoretically possible type I TAs. The toxicity of six selected putative type I toxins from this list was assessed by expressing them in *Escherichia coli* BL21(DE3) pLysS cells and observing the effects of their expression on cell growth. In general, it is assumed that the toxins are likely to exert their effects in bacterial species other than the parent ones. Both control samples showed the expected result after induction of protein expression from pET28b(+) derivatives, with the negative control cells growing normally, while cell lysis was observed in the positive control cells.

We originally intended to use the Hok type I toxin from *E. coli* as the positive control, as it is one of the best-studied type I toxins [41]. However, a BLAST search revealed that the Hok/Sok locus is already present in the genome of *E. coli* BL21(DE3) pLysS. To avoid the possibility of cross-interaction between the endogenous Sok antitoxin and the plasmid-encoded Hok toxin, we chose SrnB—a homolog of Hok that is not present in *E. coli* BL21(DE3) pLysS [9]. Comparing the effect of SrnB expression in our assay with that of Wilmaerts et al. [37], a difference in the onset of bacteriolysis can be seen. While the negative effects of SrnB expression on cell growth were observed as early as 30 min after induction in our assay, Wilmaerts et al. observed that both cell growth arrest and lysis occurred more than 120 min after induction. This is most likely due to the difference in the expression systems used, as Wilmaerts expressed SrnB from the arabinose-inducible P*_BAD_* promoter, whereas we expressed it from the IPTG-inducible T7 promoter, which is stronger compared to P*_BAD_* [42]. As for the test samples, the putative type I toxins BH695_0320 and MsoT1 were found to have a mild bacteriostatic and bacteriolytic effect, respectively, when expressed in *E. coli*, both of which occur much later than when SrnB is expressed. In contrast, no effect on cell growth was observed after the expression of the putative type I toxins BH695_0311, BH695_3336, BH695_4989, and BH695_5020. Although the results show that these four predicted toxins are not toxic to *E. coli* cells, we cannot state with certainty that they are indeed not type I toxins, as the cell toxicity assay was not performed in *M. aeruginosa*. Namely, studies have shown that some toxins exert a different effect when expressed in a heterologous organism. Jahn et al. found that the expression of BsrG and TxpA, two type I toxins from *Bacillus subtilis*, has no toxic effect in *E. coli* [43], while Ames et al. showed that the ParE type II toxin from *Pseudomonas aeruginosa* has a weaker toxic effect when expressed in *E. coli* [44]. Furthermore, overexpression from the strong inducible T7 promoter may have led to misfolding of the expressed proteins, rendering them inactive.

When considering the effect of cyanobacterial type I toxins expressed in *E. coli*, the differences in the composition of the membrane lipids should also be taken into account. Type I toxins are membrane proteins, and there may be a difference between their membrane incorporation in a typical phospholipid bilayer found in most bacteria and the glycolipid-rich membranes of cyanobacteria (reviewed in [45]). This could also explain the presence of proteins with a similar primary structure only in cyanobacteria.

To clarify whether BH695_0311, BH695_3336, BH695_4989, and BH695_5020 are physiological type I toxins, the cell toxicity assay would have to be performed in a cyanobacterium, e.g., one with established protocols for the expression of recombinant proteins, such as *Synechocystis* sp. PCC 6803 [46]. This assay could also be used to confirm the bacteriostatic and/or bacteriolytic activity of BH695_0320 and MsoT1 and to compare whether these effects are faster and stronger when expressed in cyanobacteria.

We continued our research focusing on the MsoT1/MsoA1 putative type I TA system, as MsoT1 showed a phenotype more similar to SrnB than BH695_0320. By constructing the pET28_MsoT1 plasmid, in which only the MsoT1 CDS was inserted, we were able to compare the toxic effects of MsoT1 expression in the presence and absence of the cognate MsoA1 antitoxin. In addition, pET28_SrnB/SrnC was constructed to compare the same with the SrnB/SrnC type I TA system from *E. coli*. In all three replicates of the cell toxicity assay, we observed that cells transformed with either the pET28_SrnB/SrnC or the pET28_MsoT1/MsoA1 plasmid succumbed to the toxic effects of the toxin with a slight delay compared to cells expressing the toxin only. Similar results were previously observed with the Hok/Sok system, where the killing of *E. coli* K-12 cells with Hok was slower in cells that also carried the antitoxin-encoding *sok* gene on the plasmid [7]. Therefore, we can confirm the antitoxic effect of MsoA1. Interestingly, we found that the antitoxic effect of MsoA1 is significantly weaker compared to SrnC (Figure 3B), which can be attributed to the relatively weak activity of the MsoA1 promoter in *E. coli*, as shown by the promoter activity assay with mRFP (Figure 5).

In the second cell toxicity assay, we attempted to establish a correlation between the toxic effects of MsoT1 and its expression level. Using immunodetection, we were able to confirm the synthesis of MsoT1 toxin, with the amount of the protein increasing with time after induction. Interestingly, the bands corresponding to His-tagged MsoT1 were positioned higher than expected based on the theoretical molecular mass of the protein. This can be explained by the fact that the prepared SDS-PAGE samples were not heated to 95 °C prior to electrophoresis, as this has been shown to lead to irreversible aggregation of some membrane proteins, resulting in them remaining in the stacking gel [47]. Consequently, the proteins may not have been fully denatured, so that the detergent SDS does not bind optimally to the protein, resulting in a lower net charge and slower migration during electrophoresis. In addition, it is predicted that MsoT1 contains two transmembrane helices that could form hydrophobic interactions that further prevent optimal binding of SDS to the protein—a phenomenon described by Rath et al. [48].

Although MsoT1 is predicted to be a membrane protein, most of the overexpressed protein was found in the soluble fraction, where membrane proteins are not normally found. A similar phenomenon was observed by Rawlings et al. with MmsF, MamF, and MmxF—three magnetosome membrane proteins from *Magnetospirillum magneticum*. When overexpressed under the control of the strong inducible T7 promoter in *E. coli*, all three proteins were found only in the soluble fraction. Further tests showed that the overexpressed membrane proteins assembled into water-soluble proteinosomes in which the hydrophobic regions were shielded from the surrounding aqueous environment. The authors discuss that the formation of proteinosomes could be a consequence of the artificially high levels of overexpressed proteins, while their expression in *M. magneticum* is subject to careful genetic regulation that reduces the accumulation of proteins in proteinosomes [49]. Since MsoT1 was overexpressed from the same T7 promoter in our study, it is possible that the rapid expression of MsoT1 caused the formation of similar proteinosome-like structures, explaining the abundance of MsoT1 in the soluble fraction. When comparing the band intensities of the soluble fractions and total cell lysates in Figure 3C, the cell lysate bands show higher intensities, suggesting that a small, yet significant, fraction of MsoT1 is present in the insoluble fraction and is therefore able to insert into the membrane, causing toxicity. Similar to MmsF, MamF, and MmxF, it is likely that the expression of MsoT1 under the control of its endogenous promoter in *M. aeruginosa* is also subject to tight genetic regulation that prevents the accumulation of inactive proteinosome-like aggregates.

In addition to the impairment of *E. coli* cell growth following the expression of SrnB and MsoT1 toxins, filamentous bacterial clumps appeared in the liquid media—a sign of cell lysis already observed by Nitta et al. in *E. coli* WK3 and W3110 cells [38]. Addition of the cell viability dye erythrosin B stained the clumps pink, confirming that they consisted of non-viable *E. coli* cells. Unfortunately, we were unable to observe whether SrnB and MsoT1 cause the formation of ‘ghost cells’ as observed with the Hok toxin [7], as the microscope we had available did not allow sufficiently high magnification.

To finalize our research, we tested the biological activity of the region containing the two predicted MsoA1 antitoxin promoters—P*_msoA1_*. For this purpose, we inserted a reporter protein CDS under the control of P*_msoA1_* and measured the constitutive expression of the reporter protein. To increase the reliability of the promoter assay, it was performed with two different reporter proteins—β-lactamase and mRFP.

Initially, during the antitoxin promoter activity assay with β-lactamase, bacterial growth in ampicillin was observed in all samples, even in the negative control where the β-lactamase coding region was inserted into a promoterless plasmid. This could be due to the fact that the pSB1C3 plasmid used was designed to block transcription from the “inside” of the multiple cloning region (MCS), but not transcription from the backbone of the plasmid continuing into the MCS [50]. Therefore, it is likely that there is an unannotated promoter upstream of the BioBrick prefix that allows expression of β-lactamase, leading to bacterial growth in ampicillin-containing medium.

After we redesigned the plasmids to contain two bacterial terminators directly upstream of the MCS, the negative control cells did not initially grow in ampicillin-containing media. However, after 8 h of cultivation, some replicates of the negative control began to grow in the medium containing 1 or 5 mg/mL ampicillin. A possible explanation for this is that despite the two terminator sequences, some β-lactamase expression still occurred from a cryptic promoter. Following bacterial cell death, β-lactamase would be released into the medium where it would degrade ampicillin until its concentration decreased to such an extent that the cells with minimal β-lactamase expression would begin to grow normally. This is supported by the observation that the delayed onset of bacterial growth was faster in LBC with 1 mg/mL ampicillin than in LBC with 5 mg/mL ampicillin. The cells of the test sample and of the positive control grew in all ampicillin concentrations, confirming the expression of β-lactamase from the P*_msoA1_* and P*_bla_* promoters, respectively. The slowest growth rate was observed in LBC containing 15 mg/mL ampicillin, which is consistent with the results of Škrlj et al. who found that the growth rate of bacteria containing the *bla* gene on a high-copy number plasmid, such as pSB1C3 and pSB1AC3, is only impaired in media containing 10 mg/mL ampicillin or higher [51]. The results of the antitoxin promoter activity assay with mRFP were comparable to the β-lactamase assay—cells of the test sample and the positive control showed significantly higher emission of red fluorescence compared to the negative control, confirming the expression of mRFP from the P*_msoA1_* and P*_J23119_* promoters, respectively. Interestingly, red fluorescence was also observed in the negative control cells, which again can be explained by transcription leaking from a cryptic promoter on the pSB1C3 backbone into the MCS.

The newly identified type I TA system thus consists of the MsoT1 protein toxin and the MsoA1 RNA antitoxin, which is encoded on the opposite strand. The main feature that distinguishes MsoT1 from other documented transmembrane type I toxins is the number of predicted transmembrane helices, as MsoT1 is predicted to contain two, whereas the others contain only one [15]. Interestingly, the Alpha Fold 3 predicted structure of MsoT1 (Supplementary Figure S9A) suggests that only the first membrane helix penetrates the membrane bilayer, whereas the second one would most likely be embedded in it, similar to monotopic membrane proteins. This is contrary to the TMHMM result, which predicted both membrane helices to penetrate the membrane. Since both the Alpha Fold 3 structure and the TMHMM results are only predictions, a future study focusing on the structural aspect of MsoT1 membrane binding is needed to identify the conformation of the second membrane helix. Using the MsoT1 amino acid sequence as a BLASTP query yields more than 100 hits. Most of them belong to predicted proteins from different *M. aeruginosa* strains, while others belong to cyanobacteria from different genera. This suggests that MsoT1/MsoA1 together with similar systems from other cyanobacteria may comprise a new family of type I TA systems with similar function and properties. Since both predicted isoforms of the MsoA1 antitoxin are complementary to the 3′-end of the MsoT1 toxin coding region as well as to the 3’-UTR, MsoA1 could exert its antitoxic effect either by recruiting an RNase to the formed RNA duplex, similar to the TxpA/RatA system [52], or by inducing conformational changes on the toxin mRNA after binding, leading to a sequestered ribosome binding site, as described for the BsrG/SR4 system [19].

## 4. Conclusions

In our work, we searched the genome of *M. aeruginosa* PCC 7806SL for loci with characteristics similar to the already known type I TA systems, but added several modifications in the algorithm to account for some deviations from the currently known properties of these systems in other bacterial phyla. Bioinformatic de novo analysis revealed ten potential loci, six of which we characterized in *Escherichia coli*. We found that expression of the toxins BH695_0320 and MsoT1 (BH695_4017) had a negative effect on *E. coli* cell growth, while the expression of BH695_0311, BH695_3336, BH695_4989, and BH695_5020 had no effect, raising the question of whether they are cyanobacteria-specific toxins that are non-functional in *E. coli*. Since expression of MsoT1 resulted not only in bacteriostasis but also in bacteriolysis, similar to the known SrnB type I toxin from *E. coli* assayed in parallel, we focused our attention on the putative MsoT1/MsoA1 type I TA system. Our results suggest that MsoA1 is able to delay the toxic effects of MsoT1 expression and thus acts as its cognate antitoxin. Furthermore, we directly confirmed MsoT1 expression by immunodetection and indirectly confirmed MsoA1 expression via the biological activity of its predicted constitutive promoters. Therefore, we believe that MsoT1/MsoA1 represents a novel type I TA system, making it the first type I TA system discovered in cyanobacteria and consequently the first case of a type I TA system found outside the Proteobacteria, Firmicutes, and Tenericutes phyla. Future work on the MsoT1 toxin will focus on expressing it in model cyanobacteria and comparing its toxic effects in cyanobacteria and *E. coli,* as well as determining the mechanism of MsoT1-induced toxicity. In addition, future work will also aim to detect the MsoA1 RNA directly (e.g., by northern blotting) and demonstrate its interaction with the MsoT1 mRNA.

## 5. Materials and Methods

### 5.1. Bioinformatic Analysis of the Microcystis aeruginosa PCC 7806SL Genome

#### 5.1.1. De Novo Search for Novel Putative Type I TA Loci

The nucleotide sequence of the *Microcystis aeruginosa* strain PCC 7806SL genome and the list containing amino acid sequences of all its predicted open reading frames (ORFs) were downloaded from the *M. aeruginosa* PCC 7806SL entry page on GenBank (Accession code: CP020771.1).

**Figure 6:**
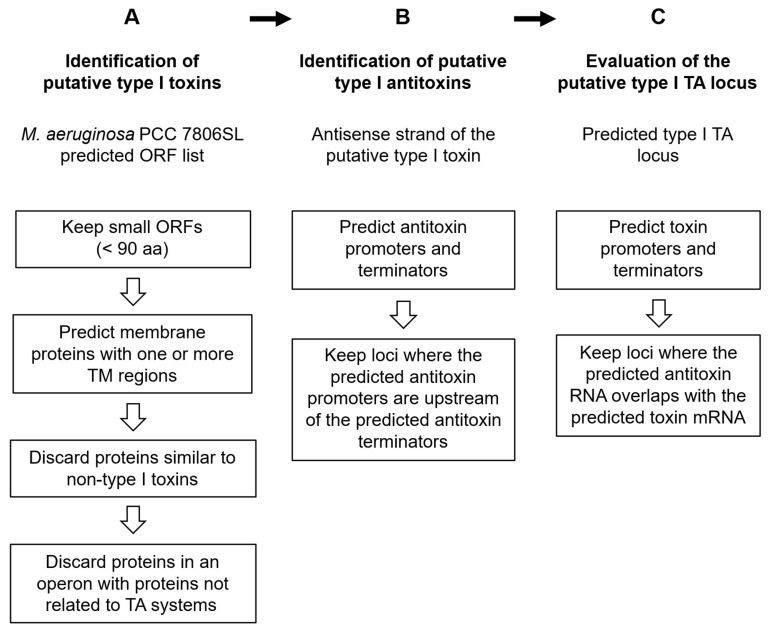
Schematic of the de novo bioinformatic search for novel putative type I TA systems in *M. aeruginosa* PCC 7806SL. The de novo search was conducted in three separate steps, starting with (**A**) the identification of putative type I toxins in the genome of *M. aeruginosa* PCC 7806SL, followed by (**B**) the identification of cognate putative type I antitoxins on the opposite strand of the predicted toxins, and concluded with (**C**) the assessment of whether the cognate putative antitoxin RNA is able to prevent translation of the putative toxin mRNA.

To identify novel putative type I toxins, we submitted the list of predicted open reading frames of *M. aeruginosa* PCC 7806SL to a series of filters similar to those used by Fozo et al. [5] to obtain proteins with characteristics similar to known type I toxins. First, we discarded all ORFs with more than 90 amino acids. Second, we analyzed the remaining small protein sequences using TMHMM (v2.0c), a transmembrane helix prediction tool [53], and removed those for which no transmembrane helices were predicted. Third, for each predicted small membrane protein, a BLAST search [54] was performed using the BLASTp algorithm (v2.12.0) to filter out those that showed similarity to proteins that are not type I toxins. Finally, in cases where the putative type I toxin protein gene was in proximity (<100 bp) to another gene on the same strand, Operon Mapper (v1.0) [55] was used to assess the possibility that the putative toxin gene was part of an operon. The putative toxin gene was excluded from the search if there was a high probability that it was part of an operon with genes not associated with TA systems (Figure 6A).

Next, we searched the antisense strand of each putative type I toxin for the cognate putative type I antitoxin gene that overlaps with the toxin gene. The analyzed region spanned from around 300 bp upstream of the toxin start codon to around 300 bp downstream of the toxin stop codon. We used bTSSfinder (v1.0) [56] and BPROM (part of the MolQuest package v2.3) [57] to predict potential antitoxin promoters, while ARNold (v1.0) [58] and FindTerm (v2.8.1) [57] were used to predict potential antitoxin terminators. When both the promoters and terminators were predicted in the correct order (promoter upstream of terminator), indicating the presence of an antisense RNA antitoxin gene, we proceeded with the analysis (Figure 6B).

Finally, to evaluate the putative type I TA locus, we determined whether the putative antitoxin RNA has a region of complementarity with the putative toxin mRNA, enabling it to inhibit the translation of the toxin. For this, we used the above-mentioned tools for the prediction of promoters and terminators of the putative type I toxin. In cases where the predicted antitoxin RNA overlapped with the predicted toxin mRNA, the pair was deemed a putative type I TA locus (Figure 6C).

#### 5.1.2. Nomenclature and Evaluation of the Predicted Putative Loci

The names of the toxins were retained as annotated in the *M. aeruginosa* PCC 7806SL genome (BH695 followed by the genome order number, i.e., numerical position of a gene in the genome), while the antitoxins were designated as MaTIA (*Microcystis aeruginosa* Type I Antitoxin) followed by the gene order number of the corresponding toxin (e.g., MaTIA_0311 is the cognate putative antitoxin of the BH695_0311 putative toxin).

The putative loci were evaluated based on the predicted promoters and terminators. If they were anticipated by both prediction tools (bTSSfinder and BPROM for promoters; ARNold and FindTerm for terminators), they were considered to exist with a high probability. In contrast, promoters and terminators that were only found by one prediction tool were considered less likely to exist.

### 5.2. Bacterial Strains

#### 5.2.1. *Escherichia coli*

For cloning of the plasmids, we used the *Escherichia coli* DH5α strain (Thermo Fisher Scientific, Waltham, MA, USA), while for the for the expression of recombinant proteins, *E. coli* BL21(DE3) and *E. coli* BL21(DE3) pLysS (Novagen, Merck, Darmstadt, Germany) were used. The *E. coli* BL21(DE3) strain (Novagen, Merck, Darmstadt, Germany) was also used to amplify the terminator region of the *rnpB M1* gene via colony PCR. The *E. coli* XL1-Blue strain (Agilent Technologies, Santa Clara, CA, USA) was used to amplify the SrnB type I toxin coding sequence and the *srnB/srnC* type I TA locus via colony PCR. The genotypes of the strains are listed in Supplementary Table S1.

#### 5.2.2. *Microcystis aeruginosa* PCC 7806SL

The *Microcystis* strain was obtained from the Pasteur Culture Collection of Cyanobacteria, where it is listed under the collection number PCC 7806SL. The genome is comprised of 5,139,339 base pairs and is predicted to contain 6991 open reading frames, according to GenBank (Accession code: CP020771.1).

### 5.3. Growth Media

Bacteria that did not carry a plasmid encoding a toxin were grown in liquid or solid Lysogeny Broth (LB) medium (1% casein peptone, 0.5% yeast extract, and 1% NaCl). Bacteria carrying a plasmid encoding a toxin were cultured in liquid or solid M9 minimal medium (1× M9 salts, 1 mM MgSO_4_, and 0.1 mM CaCl_2_), which was enriched with either 0.2% glucose, 0.1% casamino acids, and 0.2 µg/mL thiamine (denoted as M9+) or 0.5% glucose, 0.2% casamino acids, and 0.5 µg/mL thiamine (denoted as M9++).

### 5.4. Molecular Techniques

All molecular biology experiments were performed according to standard procedures and supplier recommendations. The Phusion High-Fidelity DNA polymerase was used for PCR amplification of genes from *E. coli* or *M. aeruginosa* genomes, while the DreamTaq DNA polymerase was used for colony PCR. Amplicons were digested with restriction enzymes and ligated into the plasmid with T4 DNA ligase. All enzymes were from Thermo Fisher Scientific (Waltham, MA, USA). The E.Z.N.A. Gel Extraction Kit (Omega Biotek, Norcross, GA, USA) was used to extract DNA from agarose gels, while the GeneJET Plasmid Miniprep Kit (Thermo Fisher Scientific, Waltham, MA, USA) was used for plasmid isolation. All primer sequences are listed in Supplementary Table S2.

### 5.5. Plasmids and Their Coding Regions

The pJET1.2/blunt cloning vector (Thermo Fisher Scientific, Waltham, MA, USA) was used for amplicon cloning. The pET28b(+) expression vector (Novagen, Merck, Darmstadt, Germany) was used for inducible expression of the recombinant proteins with isopropyl-1-thio-β-D-galactopyranoside (IPTG). The pSB1C3, pSB1AC3, and pSB1A2 synthetic biology vectors were used for the preparation of constructs using the RFC10 BioBrick assembly standard. They were obtained from the Registry of Standard Biological Parts (https://parts.igem.org/ (accessed on 17 June 2025)). The plasmid maps are shown in Supplementary Figures S1–S6.

#### 5.5.1. pET28_empty

The empty pET28b(+) expression vector does not contain an insert under the control of the IPTG-inducible T7 promoter, resulting only in expression of a triple tag (55 amino acids) when IPTG is added.

#### 5.5.2. pET28_0311_TA, pET28_0320_TA, pET28_3336_TA, pET28_4017_TA (pET28_MsoT1/MsoA1), pET28_4989_TA, and pET28_5020_TA

The BH695_0311/MaTIA_0311, BH695_0320/MaTIA_0320, BH695_3336/MaTIA_3336, BH695_4017/MaTIA_4017 (later renamed to MsoT1/MsoA1), BH695_4989/MaTIA_4989, and BH695_5020/MaTIA_5020 putative type I TA loci were amplified by PCR from chromosomal DNA of *Microcystis aeruginosa* strain PCC 7806SL, which was previously isolated by Marina Klemenčič from our group [33], using the corresponding primers (Supplementary Table S2). The PCR products were digested with *Nco*I and *Xho*I and inserted into pET28b(+) plasmids digested with the same restriction enzymes. The recombinant vectors lead to the expression of toxins from the IPTG-inducible T7 promoter and, on the antisense strand, expression of the putative antitoxins from their predicted constitutive promoters.

#### 5.5.3. pET28_4017_T (pET28_MsoT1)

The gene encoding the BH695_4017 (later renamed to MsoT1) putative type I toxin was amplified from the pET28_MsoT1/MsoA1 plasmid using the primers 4017H_F_NX_pET and 4017H_R_NX_pET (Supplementary Table S2). The PCR product was digested with *Nco*I and *Xho*I and inserted into the pET28b(+) plasmid, which was digested with the same restriction enzymes. The recombinant vector leads to the expression of the C-terminally His-tagged MsoT1 from the IPTG-inducible T7 promoter.

#### 5.5.4. pET28_SrnB

The gene encoding SrnB—a type I toxin from plasmid F of *Escherichia coli* [9]—was amplified from *E. coli* XL1-Blue using the primers SrnB_F_NX and SrnB_R_NX (Supplementary Table S2). The PCR product was digested with *Nco*I and *Xho*I and inserted into the pET28b(+) plasmid, which was digested with the same restriction enzymes. The recombinant vector leads to the expression of the SrnB type I toxin from the IPTG-inducible T7 promoter.

#### 5.5.5. pET28_SrnB/SrnC

The *srnB/srnC* type I TA locus was amplified from *E. coli* XL1-Blue using the primers *srnB/srnC*_F_XX and SrnB_R_NX (Supplementary Table S2). The PCR product was digested with *Xba*I and *Xho*I and inserted into the pET28b(+) plasmid, which was digested with the same restriction enzymes. The recombinant vector leads to the expression of the SrnB type I toxin from the IPTG-inducible T7 promoter and to the expression of its cognate SrnC type I antitoxin from its endogenous constitutive P*_srnC_* promoter present on the antisense strand.

#### 5.5.6. pSB1C3_RBS_AmpR

The sequence coding for the β-lactamase enzyme was amplified from the pJET1.2/blunt plasmid using the primers RBS-AmpR_BB_F and AmpR_BB_R (Supplementary Table S2). The RBS-AmpR_BB_F primer was designed to contain a ribosome binding site in the overhang. The PCR product was digested with *Eco*RI and *Pst*I and inserted into the pSB1C3 plasmid, which was digested with the same restriction enzymes. The recombinant vector lacks a promoter, resulting in no expression of β-lactamase.

#### 5.5.7. pSB1C3_TT_RBS_AmpR

The *rnpB M1* terminator was amplified from *E. coli* BL21(DE3) using the primers Ter-rnpB-v2_F and rnpB-Ter_BB_R (Supplementary Table S2). The PCR product was digested with *Eco*RI and *Spe*I and inserted into the pSB1C3_RBS_AmpR plasmid, which was digested with *Eco*RI and *Xba*I. The recombinant vector lacks a promoter, resulting in no expression of β-lactamase. In addition, the two terminator sequences upstream of the RBS prevent transcription from potential cryptic promoters upstream on the vector backbone.

#### 5.5.8. pSB1C3_P*_msoA1_*_RBS_AmpR

The two predicted promoters of the MsoA1 putative type I antitoxin (P*_msoA1_*) were amplified by PCR from the isolated chromosomal DNA of *M. aeruginosa* strain PCC 7806SL using the primers anti4017_BB_F and anti4017_BB_R (Supplementary Table S2). The PCR product was digested with *Eco*RI and *Spe*I and inserted into the pSB1C3_RBS_AmpR plasmid, which was digested with *Eco*RI and *Xba*I. The recombinant vector leads to the expression of β-lactamase from the predicted constitutive P*_msoA1_* promoters.

#### 5.5.9. pSB1C3_TT_P*_msoA1_*_RBS_AmpR

The *rnpB M1* terminator was amplified from *E. coli* BL21(DE3) using the primers Ter-rnpB-v2_F and rnpB-Ter_BB_R (Supplementary Table S2). The PCR product was digested with *Eco*RI and *Spe*I and inserted into the pSB1C3_P*_msoA1_*_RBS_AmpR plasmid, which was digested with *Eco*RI and *Xba*I. The recombinant vector leads to the expression of β-lactamase from the predicted constitutive P*_msoA1_* promoters. Furthermore, the two terminator sequences upstream of the P*_msoA1_* promoters prevent transcription from potential cryptic promoters upstream on the vector backbone.

#### 5.5.10. pSB1AC3_empty

The empty pSB1AC3 synthetic biology vector contains the *bla* gene encoding the β-lactamase enzyme on its backbone, leading to expression of the enzyme from its endogenous constitutive P*_bla_* promoter.

#### 5.5.11. pSB1C3_K516032

This vector is based on the pSB1C3 synthetic biology vector. It contains the BioBrick BBa_K516032 (Registry of Standard Biological Parts), which consists of a medium efficiency RBS (BBa_B0032), the coding sequence (CDS) of the monomeric red fluorescent protein (mRFP) (BBa_E1010) and a double terminator (BBa_B0015). The recombinant vector lacks a promoter, resulting in no expression of mRFP.

#### 5.5.12. pSB1C3_P*_msoA1_*_K516032

The two predicted promoters of the MsoA1 putative type I antitoxin (P*_msoA1_*) were amplified by PCR from the isolated chromosomal DNA of *M. aeruginosa* strain PCC 7806SL using the primers anti4017_BB_F and anti4017_BB_R (Supplementary Table S2). The PCR product was digested with *Eco*RI and *Spe*I and inserted into the pSB1C3_K516032 plasmid, which was digested with *Eco*RI and *Xba*I. The recombinant vector leads to the expression of mRFP from the predicted constitutive P*_msoA1_* promoters.

#### 5.5.13. pSB1A2_J23119_K516032

The pSB1C3_K516032 vector was digested with *Xba*I and *Pst*I. The BBa_K516032 BioBrick was then inserted into the pSB1A2_J23119 vector, which was digested with *Spe*I and *Pst*I. The BBa_J23119 BioBrick (Registry of Standard Biological Parts) consists of a strong constitutive promoter. The recombinant vector leads to the expression of mRFP from the constitutive P*_J23119_* promoter.

### 5.6. Cell Toxicity Assay

Overnight cultures of *Escherichia coli* BL21(DE3) pLysS cells transformed with the corresponding plasmids were diluted to OD_600_ = 0.05 in M9+ or M9++ medium containing 50 µg/mL kanamycin and 25 μg/mL chloramphenicol and grown in shaking flasks on an orbital shaker at 37 °C and 120 rpm. The OD_600_ values were measured using the UV-1600PC UV/Vis spectrophotometer (VWR, Radnor, PA, USA). When the OD_600_ values reached between 0.4 and 0.6, toxin expression was induced by adding IPTG at a final concentration of 0.5 mM. After induction, cultures were diluted prior to OD_600_ measurement so that the measured optical density did not exceed 1.0. The actual OD_600_ was then determined by multiplying the measured value by the dilution coefficient. Before induction, the OD_600_ was measured every 30 min, while after induction it was measured every 30–60 min. Cell toxicity was qualitatively assessed by comparing growth curves. The raw data is shown in Supplementary Files S1 and S2.

### 5.7. Promoter Activity Assays

#### 5.7.1. Antitoxin Promoter Activity Assay with β-Lactamase

Overnight cultures of *Escherichia coli* BL21(DE3) cells transformed with the corresponding plasmids were diluted to OD_600_ = 0.02 in LB medium containing 25 µg/mL chloramphenicol and either 0, 100, 200, 300, 1000, 5000, or 15,000 µg/mL ampicillin. Cells were grown in 48-well microtiter plates (Greiner Bio-One, Kremsmünster, Austria) in the BioTek Synergy H1 microplate reader (Agilent Technologies, Santa Clara, CA, USA) at 37 °C and 160 rpm. The microplate reader program was set to measure OD_600_ every 15 min for the first 2 h, and then every 60 min for 10 h. Promoter activity was assessed by comparing growth curves obtained in the presence of increasing concentrations of ampicillin. The raw data is shown in Supplementary Files S3 and S5.

#### 5.7.2. Antitoxin Promoter Activity Assay with mRFP

Overnight cultures of *Escherichia coli* BL21(DE3) cells transformed with the corresponding plasmids were diluted to OD_600_ = 0.02 in LB medium containing either 25 µg/mL chloramphenicol for pSB1C3-based plasmids or 100 µg/mL ampicillin for pSB1A2-based plasmids. Cells were grown in 48-well microtiter plates (Greiner Bio-One, Kremsmünster, Austria) in the BioTek Synergy H1 microplate reader (Agilent Technologies, Santa Clara, CA, USA) at 37 °C and 160 rpm. The microplate reader program was set to measure OD_600_ and fluorescence (λ_excitation_ = 570 nm, λ_emission_ = 620 nm) every 15 min for the first 2 h, and then every 60 min for 10 h. The absolute fluorescence units (AFUs) were normalized by dividing them by the OD_600_ values. Relative promoter activity was determined by comparing fluorescence curves of different constructs. The raw data is shown in Supplementary File S4.

### 5.8. Western Blotting and Immunodetection

For detection of the C-terminally His-tagged MsoT1, an aliquot of *Escherichia coli* BL21(DE3) pLysS cells transformed with the pET28_MsoT1 plasmid was harvested 1, 2, 4, and 6 h after induction with IPTG. Cells were resuspended in ultrapure water (MilliQ, Merck Millipore, Darmstadt, Germany) and sonicated on ice for 3 × 10 s at 80% power on the UP100H device (Hielscher, Teltow, Germany). The obtained cell lysate was electrophoretically separated on a 20% Tris-tricine polyacrylamide gel and then blotted onto a polyvinylidene fluoride (PVDF) membrane. In separate experiments, cell lysates were centrifuged at 17,000× *g* for 5 min prior to electrophoresis to separate the soluble fraction (supernatant) from the insoluble fraction (pellet). The membrane was blocked for 1 h in 1× TBST buffer containing 5% (*w*/*v*) skim milk powder (Merck Millipore, Darmstadt, Germany) and then incubated for 1 h in 1× TBST buffer containing 1% (*w*/*v*) powdered milk and a 1:1000 dilution of mouse horseradish peroxidase-conjugated anti-His antibodies (Roche, Basel, Switzerland). The membrane was then thoroughly washed with 1× TBST buffer and developed with Clarity Western ECL Substrate (Bio-Rad, Hercules, CA, USA). The chemiluminescence was recorded with the ChemiDoc imager (Bio-Rad, Hercules, CA, USA).

### 5.9. Erythrosin B Staining of Non-Viable Bacterial Cells

To estimate the proportion of dead bacterial cells, aliquots of *Escherichia coli* BL21(DE3) pLysS cells transformed with the pET28_empty, pET28_SrnB/SrnC, pET28_SrnB, pET28_MsoT1/MsoA1, or pET28_MsoT1 plasmids were taken at the end of the cell toxicity assay. Each aliquot was mixed with a 1:1 ratio of 0.8% (*w*/*v*) erythrosin B and incubated for 5 min at room temperature. After incubation, a total of 5 µL of the cell-dye mixture was taken to prepare wet mounts for brightfield microscopy. Imaging was performed using a Zeiss Primo Star microscope with an AxioCam ERc 5s camera (Zeiss, Oberkochen, Germany).

### 5.10. Statistical Analysis

For assays that were repeated at least three times, the arithmetic mean was used to calculate the average value of the measurement, while the accuracy of the mean value was assessed using standard error.

## Figures and Tables

**Table 1:** Predicted putative type I toxins in the *M. aeruginosa* PCC 7806SL genome (GenBank accession: CP020771.1) with their genome positions, strand, length and number of transmembrane (TM) regions.

Name	GenBank Accession	Start	End	Strand	Length (aa)	TM Regions
BH695_0311	ARI79592.1	289,680	289,489	−1	63	1
BH695_0320	ARI79601.1	299,411	299,551	1	46	1
BH695_1184	ARI80465.1	1,096,632	1,096,486	−1	48	1
BH695_1248	ARI80529.1	1,152,754	1,152,629	−1	41	1
BH695_3336	ARI82615.1	3,177,614	3,177,757	1	47	1
BH695_3390	ARI82669.1	3,230,378	3,230,259	−1	39	1
BH695_4017	ARI83296.1	3,792,667	3,792,476	−1	63	2
BH695_4253	ARI83532.1	4,016,545	4,016,411	−1	44	1
BH695_4989	ARI84268.1	4,737,004	4,737,249	1	81	2
BH695_5020	ARI84299.1	4,769,107	4,768,946	−1	53	1

**Table 2:** Predicted cognate putative type I antitoxins from the *M. aeruginosa* PCC 7806SL genome (GenBank accession: CP020771.1) with their genomic position, strand, length, and complementarity region with the corresponding toxin mRNA (region of the predicted type I toxin mRNA to which the predicted cognate type I antitoxin is complementary). In cases where multiple promoters were predicted, all possible antitoxin isoforms are listed.

Name	Isoform	Start	End	Strand	Length (nt)	Toxin mRNA Complementarity
MaTIA_0311	1	289,493	289,535	1	43	CDS
	2	289,482	289,535	1	54	CDS, 3′-UTR
MaTIA_0320	1	299,493	299,423	−1	71	CDS
	2	299,517	299,423	−1	95	CDS
	3	299,574	299,423	−1	152	CDS, 3′-UTR
MaTIA_1184	1	1,096,424	1,096,466	1	43	3′-UTR
	2	1,096,327	1,096,466	1	140	3′-UTR
MaTIA_1248	-	1,152,693	1,152,843	1	151	CDS, 5′-UTR
MaTIA_3336	1	3,177,829	3,177,749	−1	81	CDS, 3′-UTR
	2	3,177,945	3,177,749	−1	197	CDS, 3′-UTR
MaT1A_3390	1	3,230,318	3,230,458	1	141	CDS, 5′-UTR
	2	3,230,304	3,230,458	1	155	CDS, 5′-UTR
MaTIA_4017	1	3,792,450	3,792,505	1	56	CDS, 3′-UTR
	2	3,792,421	3,792,505	1	85	CDS, 3′-UTR
MaTIA_4253	-	4,016,466	4,016,717	1	252	CDS, 5′-UTR
MaTIA_4989	-	4,737,319	4,737,227	−1	93	CDS, 3′-UTR
MaTIA_5020	1	4,768,937	4,768,986	1	50	CDS, 3′-UTR
	2	4,768,892	4,768,986	1	95	CDS, 3′-UTR

## Data Availability

The original contributions presented in this study are included in the article/Supplementary Materials. Further inquiries can be directed to the corresponding author.

## Supplementary Materials

The following supporting information can be downloaded at: https://www.mdpi.com/article/10.3390/toxins17080360/s1, Figure S1: pJET1.2/blunt plasmid map; Figure S2: pET-28(+) plasmid map; Figure S3: pSB1C3 plasmid map; Figure S4: pSB1AC3 plasmid map; Figure S5: pSB1C3-BBa_K516032 plasmid map; Figure S6: pSB1A2-BBa_J23119 plasmid map; Figure S7: Microscopy images of *E. coli* cells treated with erythrosin B; Figure S8: Growth of *E. coli* BL21(DE3) cells expressing β-lactamase from the P*_bla_* and P*_msoA1_* constitutive promoters in media with or without ampicillin; Figure S9: Predicted structures of the MsoT1/MsoA1 Toxin–Antitoxin pair; File S1: Data for Figure 2; File S2: Data for Figure 3; File S3: Data for Figure 4; File S4: Data for Figure 5; File S5: Data for Supplementary Figure S8; Table S1: Genotypes of *Escherichia coli* strains used in the study; Table S2: Primers used in the study.
