# Supplementary material for: Microcystis aeruginosa msoT1/msoA1 Locus Displays Features of a Type I Toxin–Antitoxin System
Source: Toxins (Basel). 2025 Jul 22;17(8):360. doi: 10.3390/toxins17080360 (PMC12390103; doi:10.3390/toxins17080360)
Supplement: Supplementary file 1 [file toxins-17-00360-s001.zip › Supplementary_Tables_and_Figures.pdf]

Supplementary Materials: *Microcystis aeruginosa msoT1/msoA1* Locus Displays Features of a Type I Toxin–Antitoxin System

Matija Ruparčič and Marko Dolinar

Table S1. Genotypes of *Escherichia coli* strains used in the study.

E. coli strain	**Genotype**
BL21(DE3)	F^-*omp*T *hsd*SB (r_B^-, m_B^-) *gal dcm* (DE3)
BL21(DE3) pLysS	F^-*omp*T *hsd*SB (r_B^-, m_B^-) *gal dcm* (DE3) pLysS(CamR)
DH5α	F^- φ80*lac*ZΔM15 Δ(*lac*ZYA-*arg*F)U169 *rec*A1 *end*A1 *hsd*R17(r_K^-, m_K^+) *pho*A *sup*E44 λ^-*thi*-1 *gyr*A96 *rel*A1
XL1-Blue	*end*A1 *gyr*A96(*nal*R) *thi*-1 *rec*A1 *rel*A1 *lac gln*V44 F'[::Tn10 *pro*AB$^+$ *lac*Iq Δ(*lac*Z)M15] *hsd*R17(r_K^- m_K^+)

Table S2. Primers used in the study. The restriction sites are underlined. Due to the sequence of the *Nco*I restriction site, some primers introduced additional amino acids to the N-terminal of the protein.

**Primer name**	**Sequence (5′ → 3′)**	**Additional aa at N-terminus**
0311_F_NX	ATACCATGGGTCTCTTAAAGCTACTGGAC	Gly
0311_R_NX	TATCTCGAGGGTGACTTTGGAAGAAG	
0320_F_NX	GTACCATGGCTTGTTTCACAAAAC	
0320_R_NX	CTACTCGAGGGAATTAAGGGAAAATCACC	
3336_F_NX	GTGCCATGGGGAATTCTGCTAAAATTATCG	Gly
3336_R_NX	TCTCTCGAGTTTCCGACAGTTAACC	
4017_F_NX	ATACCATGGGCATGAGACCCGTT	Gly–Met
4017_R_NX	TGTCTCGAGTTGATTGCCATCGATAC	
4017H_F_NX_pET	ATACCATGGGCATGAG	Gly–Met
4017H_R_NX_pET	ATACTCGAGATATTTGCCCTTGAGG	
4989_F_NX	TATCCATGGGAGTGGGGGGTCTGGTT	Gly–Val
4989_R_NX	CTTCTCGAGCTTGGATGACCATTCGTCTG	
5020_F_NX	ATCCCATGGATCCATGTTTAGCG	
5020_R_NX	ATACTCGAGGTCTCCATAGTTCAATCGG	
SrnB_F_NX	ATACCATGGGGAAGTACCTTAACACTACTGA TTG	Gly
SrnB_R_NX	GTGCTCGAGTTACTTCCGTGCTTCGTAGG	
srnB/*srnC*_F_XX	GTATCTAGACCTTTCTGGTTCAGGCAAG	
anti4017_BB_F	ATCTCGAATTCGCGGCCGCTTCTAGAGAAAT AGCAGTTTCCCGGTCTC	
anti4017_BB_R	ATACTGCAGCGGCCGCTACTAGTAAACATAT TGCCAAGGTTGCG	

RBS-AmpR_BB_F	GCATCTAGAGAAGGAGATATACCATGAGTATTCAACATTTCCG	
AmpR_BB_R	CTACTGCAGCGGCCGCTACTAGTATTATTACCAATGCTTAATCAGTG	
Ter-rnpB-v2_F	ATCTCGAATTCGCGGCCGCTTCTAGAGAGCGATTGCTGGCCTAGATG	
rnpB-Ter_BB_R	ATACTGCAGCGGCCGCTACTAGTACTTTCTTTTGGGTATAGTGTCGTG	

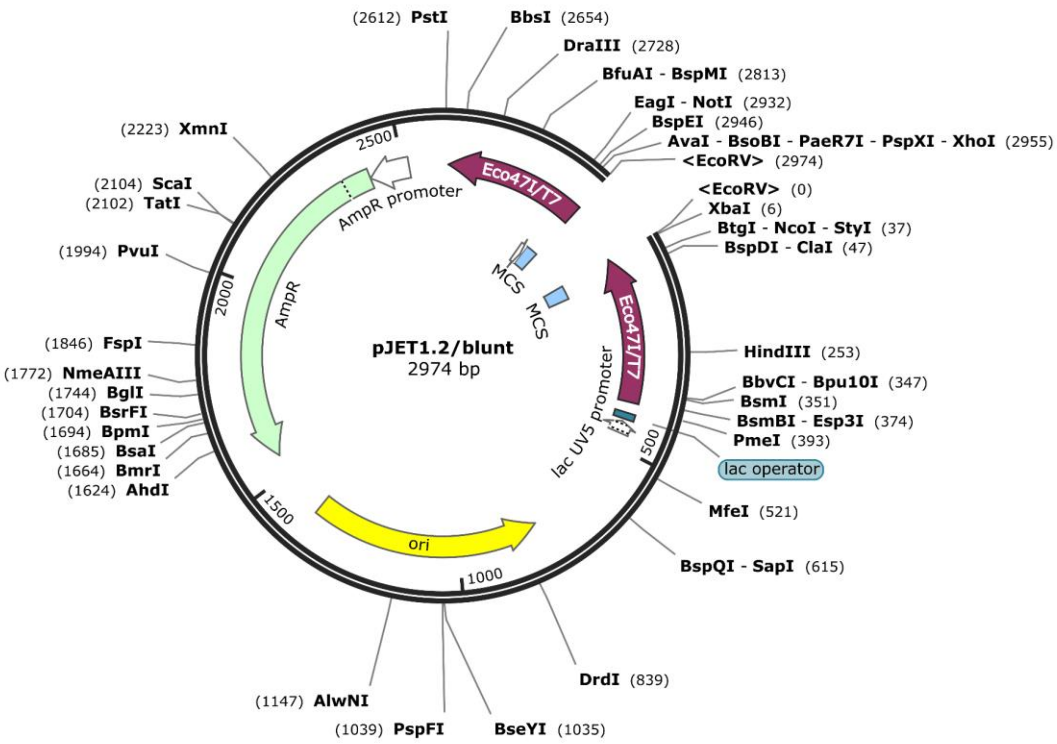

Figure S1. pJET1.2/blunt plasmid map. Shown are the bacterial origin of replication (ori), *lac* UV5 promoter, *lac* operator, *Eco*47I restriction enzyme gene, multiple cloning site (MCS), gene encoding the β-lactamase enzyme (AmpR) which provides resistance to the antibiotic ampicillin, and its promoter. The restriction enzyme sites are shown in bold. The plasmid is linearized so that the *Eco*47I gene is split, preventing the growth of cells harboring pJET1.2 plasmids with no insert due to the lethal activity of the intact *Eco*47I enzyme. The map was prepared in SnapGene v8.1.1.

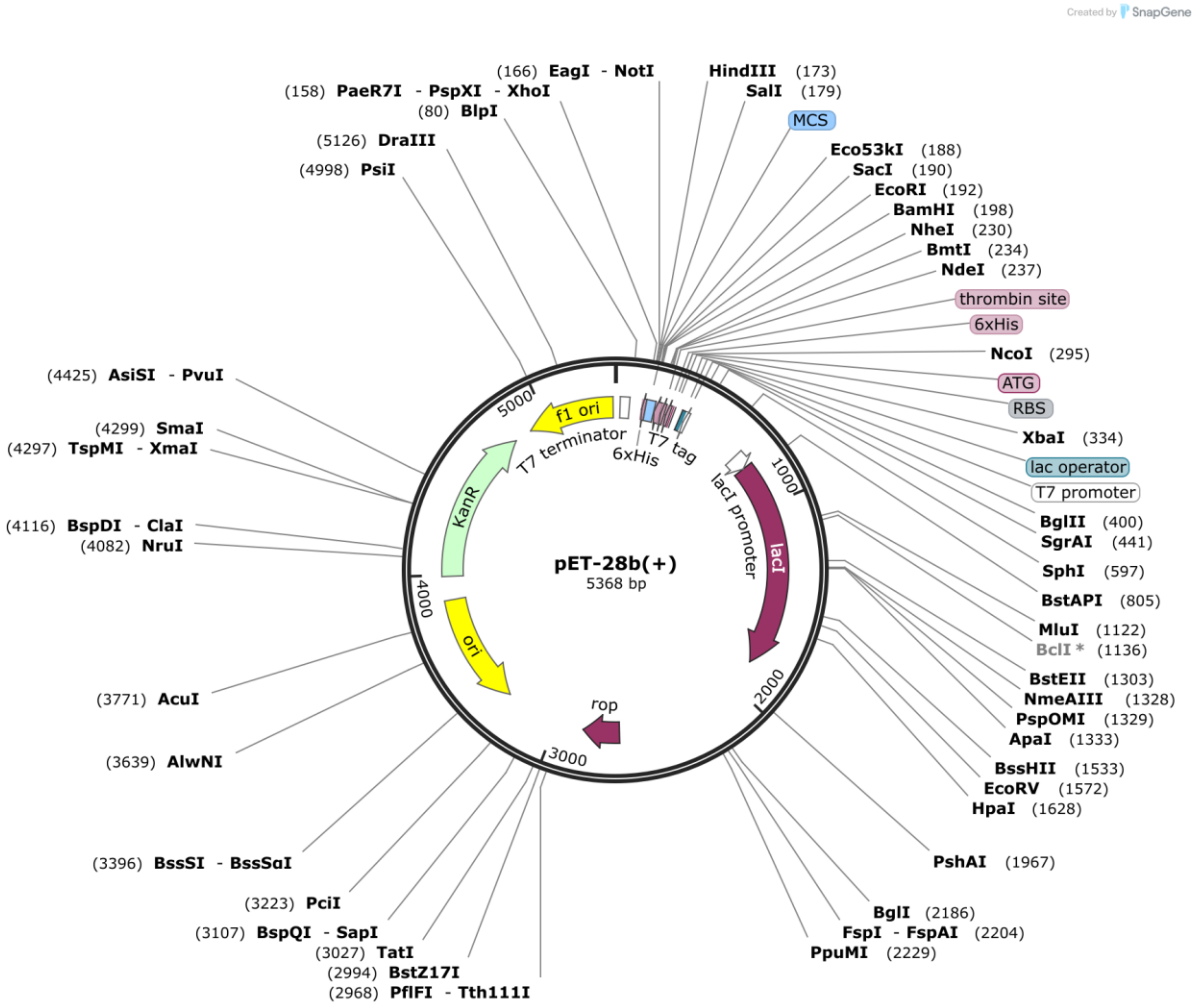

Figure S2. pET-28(+) plasmid map. Shown are the bacterial origin of replication (ori), f1 bacteriophage origin of replication (f1 ori), Rop protein gene, gene encoding the aminoglycoside 3'-phosphotransferase enzyme (KanR), which provides resistance to the antibiotic kanamycin, gene encoding the LacI repressor and its promoter, T7 promoter, *lac* operator, ribosome binding site (RBS), start codon (ATG), His-tag (6xHis), T7 tag, thrombine cleavage site, multiple cloning site (MCS), and T7 terminator. The restriction enzyme sites are shown in bold. The map was prepared in SnapGene v8.1.1.

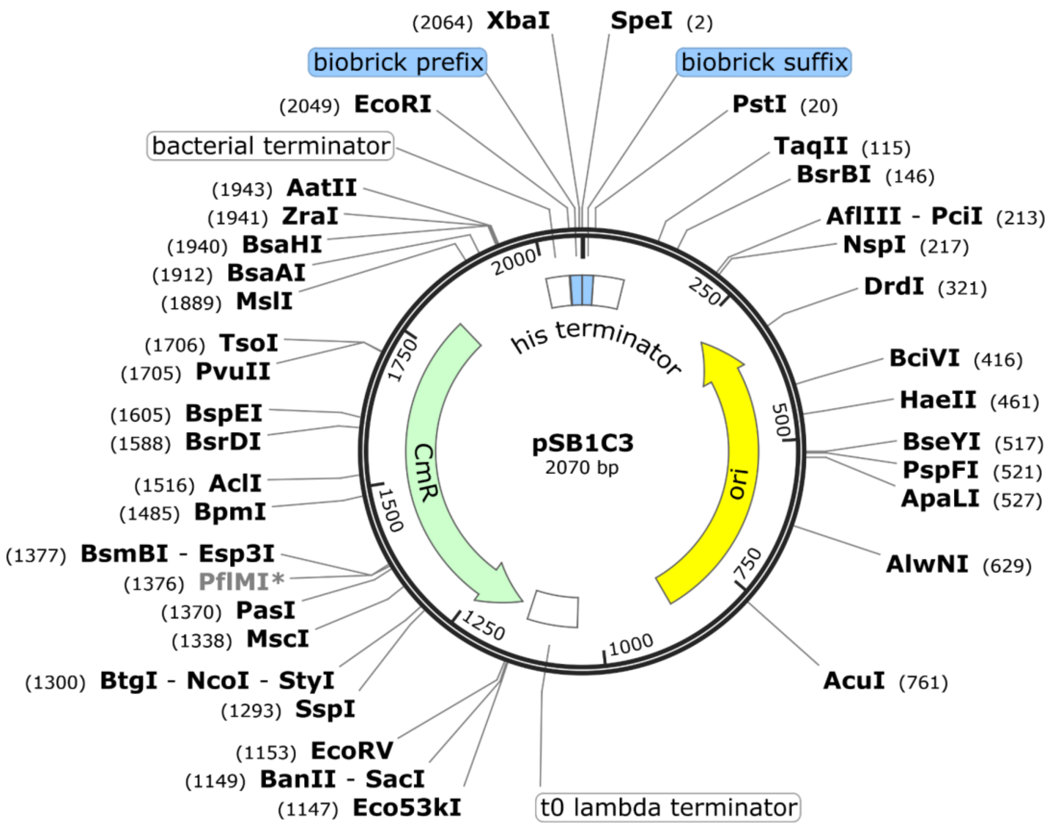

Figure S3. pSB1C3 plasmid map. Shown are the bacterial origin of replication (ori), gene encoding the chloramphenicol O-acetyltransferase enzyme (CmR), which provides resistance to the antibiotic chloramphenicol, bacteriophage t0 terminator, *his* operon terminator, unnamed bacterial terminator, biobrick prefix and biobrick suffix. The restriction enzyme sites are shown in bold. The map was prepared in SnapGene v8.1.1.

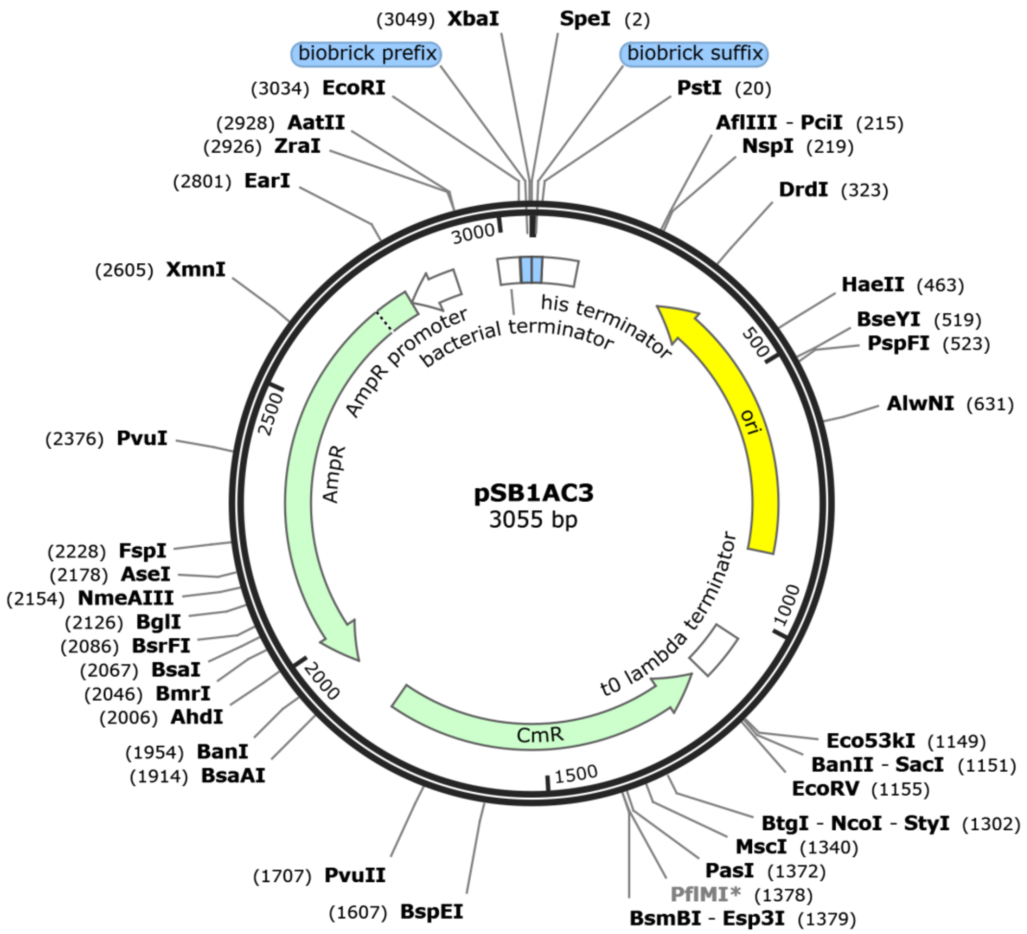

Figure S4. pSB1AC3 plasmid map. Shown are the bacterial origin of replication (ori), gene encoding the chloramphenicol O-acetyltransferase enzyme (CmR), which provides resistance to the antibiotic chloramphenicol, gene encoding the β-lactamase enzyme (AmpR) which provides resistance to the antibiotic ampicillin and its promoter, bacteriophage t0 terminator, *his* operon terminator, unnamed bacterial terminator, biobrick prefix and biobrick suffix. The restriction enzyme sites are shown in bold. The map was prepared in SnapGene v8.1.1.

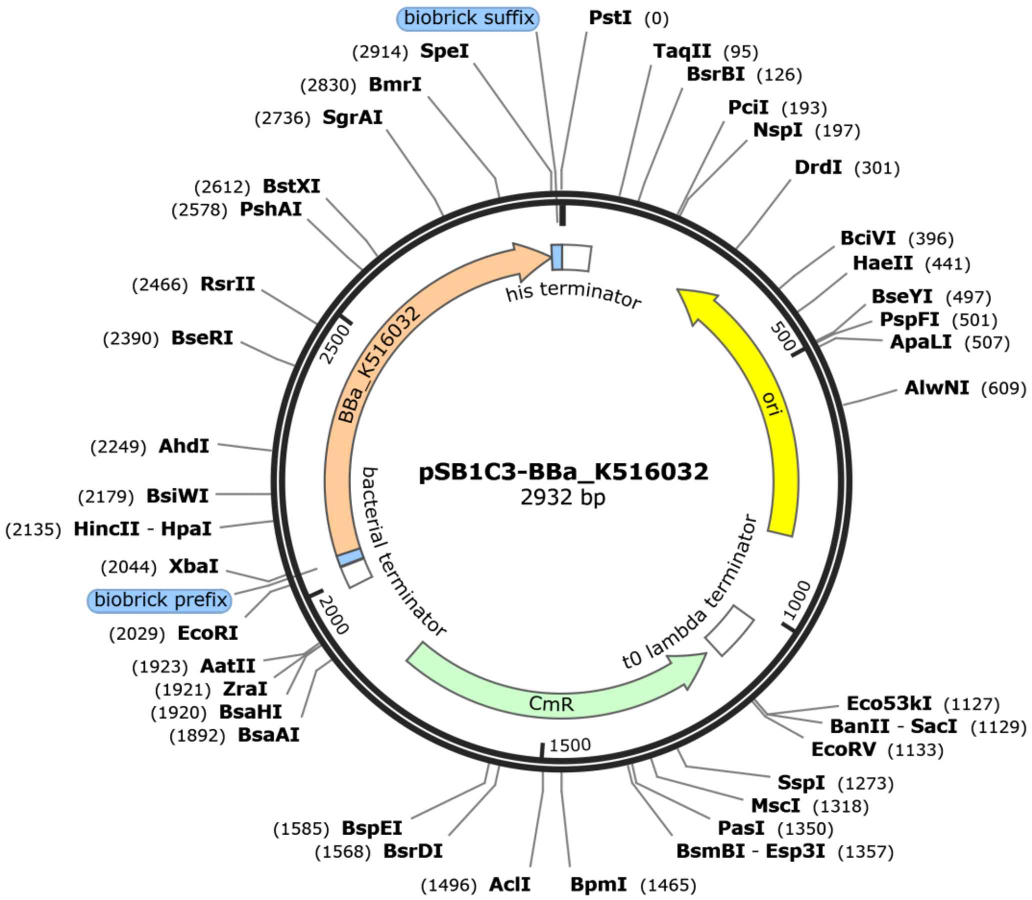

Figure S5. pSB1C3-BBa_K516032 plasmid map. Shown are the bacterial origin of replication (ori), gene encoding the chloramphenicol O-acetyltransferase enzyme (CmR), which provides resistance to the antibiotic chloramphenicol, bacteriophage t0 terminator, *his* operon terminator, unnamed bacterial terminator, biobrick prefix, biobrick suffix, and the pre-inserted BBa_K51603 biobrick. The restriction enzyme sites are shown in bold. The map was prepared in SnapGene v8.1.1.

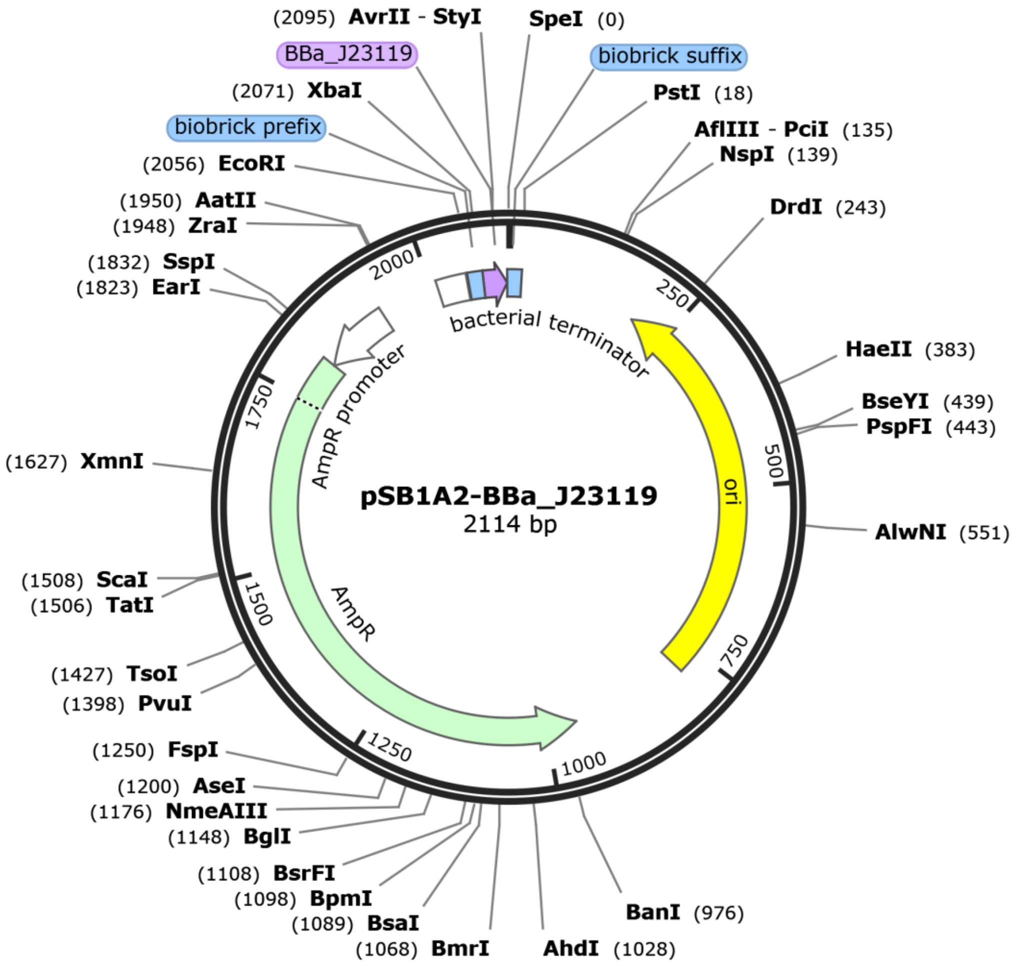

Figure S6. pSB1A2-BBa_J23119 plasmid map. Shown are the bacterial origin of replication (ori), gene encoding the β-lactamase enzyme (AmpR) which provides resistance to the antibiotic ampicillin and its promoter, bacteriophage t0 terminator, unnamed bacterial terminator, biobrick prefix, biobrick suffix, and the pre-inserted BBa_J23119 biobrick. The restriction enzyme sites are shown in bold. The map was prepared in SnapGene v8.1.1.

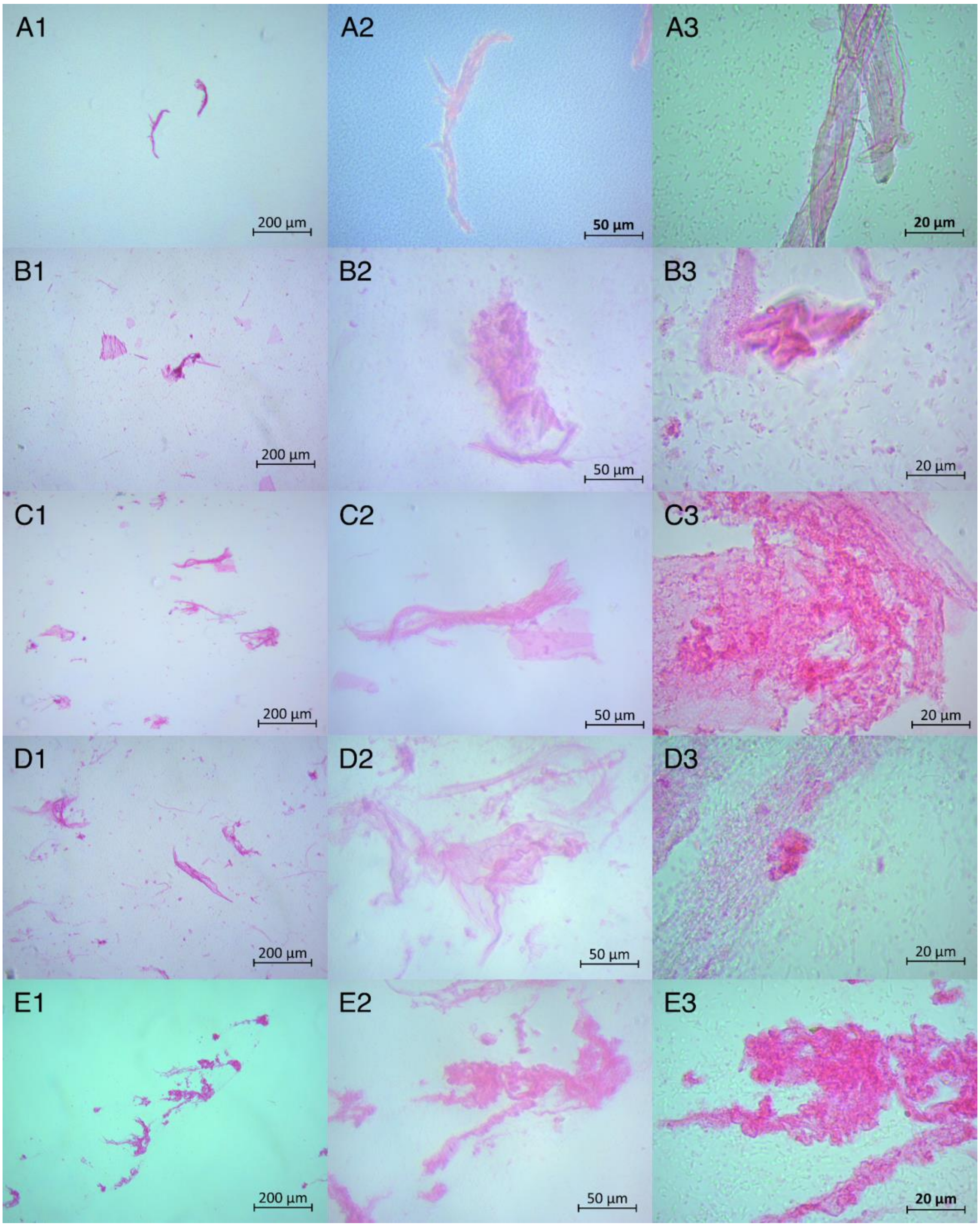

Figure S7. Microscopy images of *E. coli* cells treated with erythrosin B. Six hours after the addition of IPTG, aliquots of *E. coli* BL21(DE3) pLysS harboring the **(A)** pET28_empty, **(B)** pET28_SrnB/SrnC, **(C)** pET28_SrnC, **(D)** pET28_MsoT1/MsoA1 and **(E)** pET28_MsoT1 plasmids were mixed in a 1:1 ratio with 0.8% (w/v) erythrosin B and incubated at room temperature for 5 minutes. Afterwards, 5 µl of the mixture was taken to prepare wet mounts for brightfield microscopy. The images were taken at 100x (numbers 1), 400x (numbers 2), and 1000x (numbers 3) amplification.

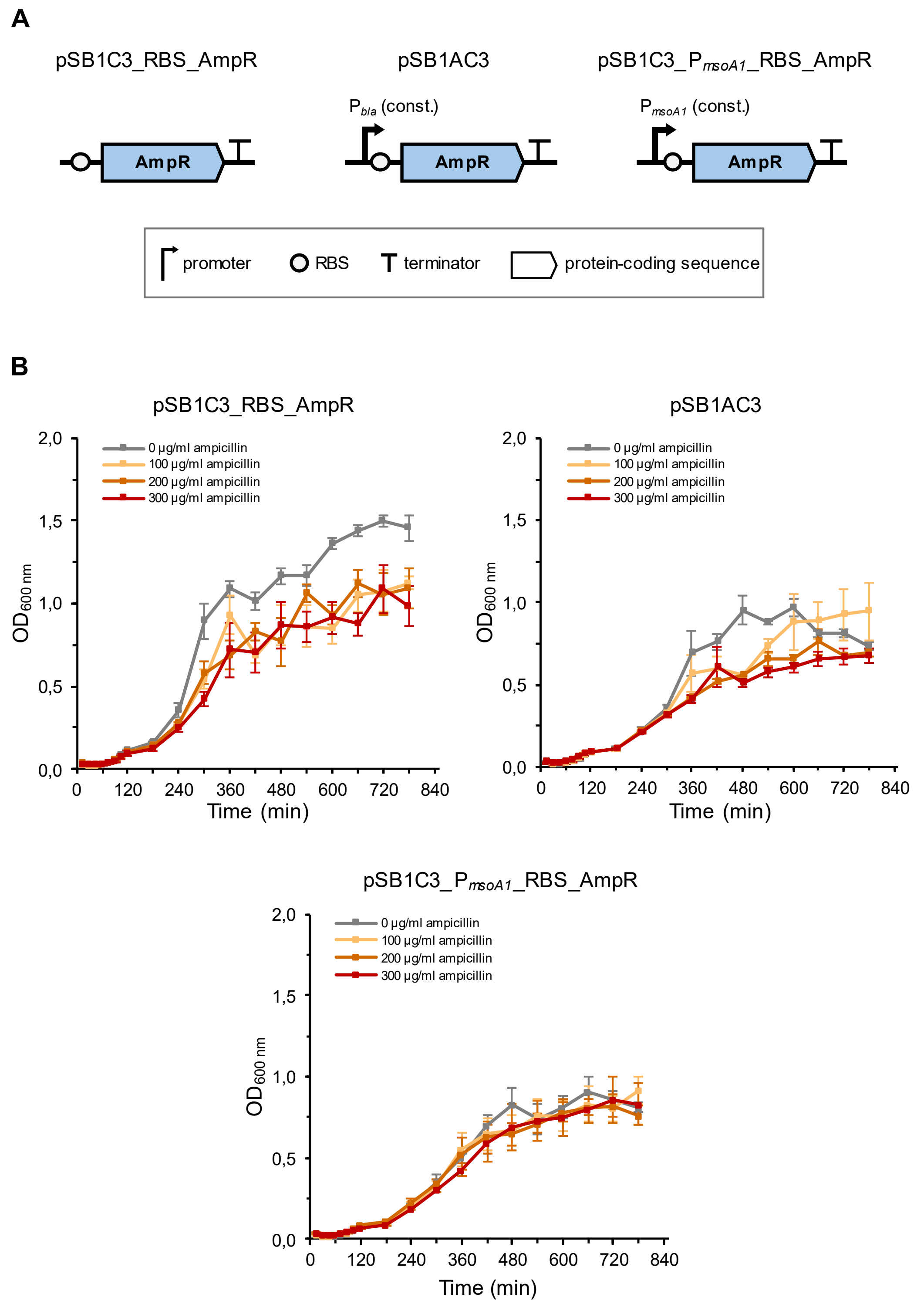

Figure S8. Growth of *E. coli* BL21(DE3) cells expressing β-lactamase from the P*bla* and P*msoA1* constitutive promoters in media with or without ampicillin. **(A)** Schematic representation of the plasmid constructs used. For the negative control, the RBS and CDS of β-lactamase were inserted in the pSB1C3 plasmid under the control of no promoter. For the positive control, the pSB1AC3 plasmid was used, where β-lactamase is under the control of its P*bla* endogenous constitutive promoter. For the test

sample, the RBS and CDS of β-lactamase were inserted in the pSB1C3 plasmid under the control of the P_{msoA1} predicted constitutive promoters. **(B)** *E. coli* BL21(DE3) transformed with the plasmids were grown in liquid LBC media at 37 °C. The media were supplemented with the antibiotic ampicillin to a final concentration of 0, 100, 200 or 300 μg/ml. The expression of β-lactamase was evaluated by monitoring cell growth in media containing ampicillin. Values represent mean ± SE of 4 sets of measurements (2 biological replicates in duplicates) for pSB1C3_RBS_AmpR and pSB1C3_P_{msoA1}_RBS_AmpR and 2 sets of measurements (1 biological replicate in duplicates) for pSB1AC3.

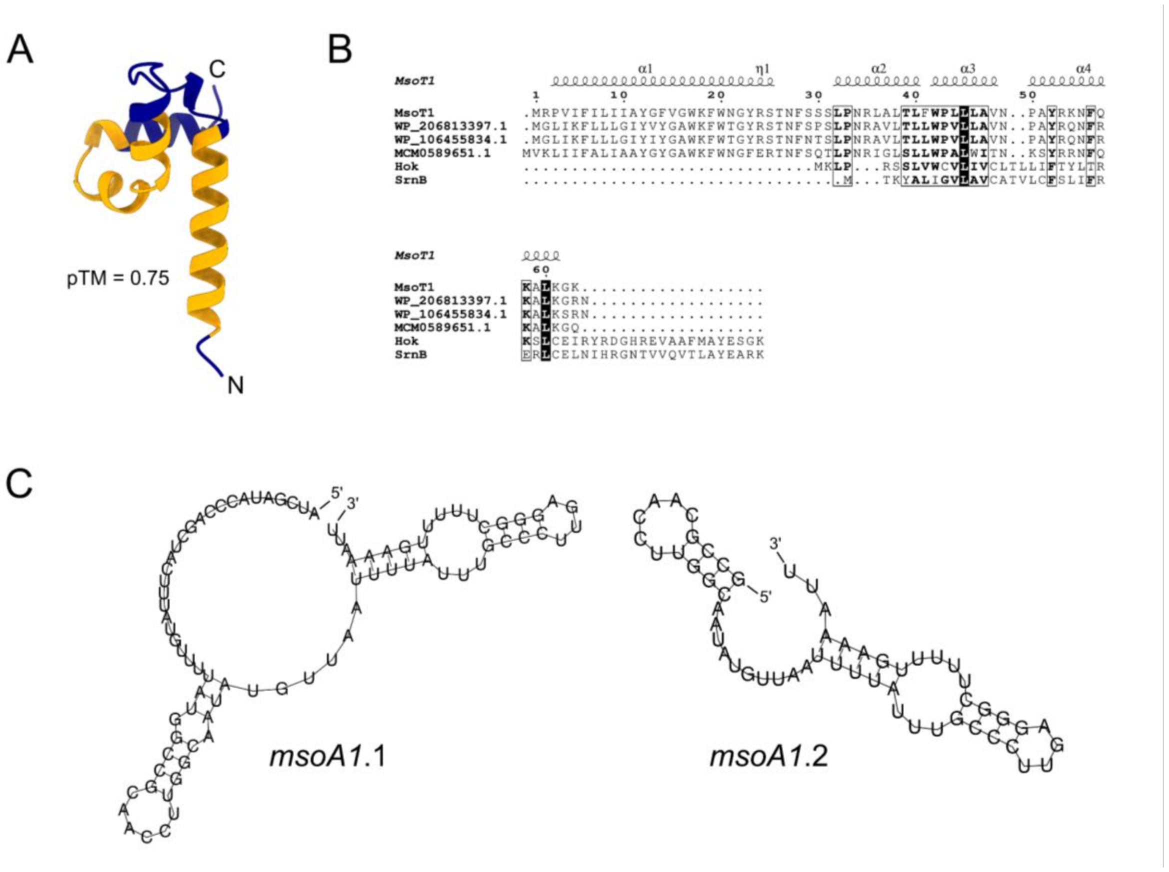

Figure S9. Predicted structures of the MsoT1/MsoA1 Toxin–Antitoxin pair. **(A)** Alpha Fold 3 [1] predicted structure of the MsoT1 type I toxin. The part of the sequence which was predicted to be inserted into the membrane by TMHMM (v2.0c) is colored orange. **(B)** Amino acid sequence alignment of MsoT1 with the Hok and SrnB type I toxins from *Escherichia coli* and the top three non-*Microcystis* hits obtained from BLASTp (v2.12.0), belonging to *Chroococcus sp.* FPU101 (WP_206813397.1), *Aphanothece hegewaldii* (WP_106455834.1), and *Gloeotrichia echinulata* DEX184 (MCM0589651.1). The secondary structure of the MsoT1 Alpha Fold 3 model is shown. The sequences were aligned with ClustalOmega (v1.2.4) and the alignment image was generated with ESPript (v3.0). **(C)** Predicted secondary structures of the two predicted MsoA1 antitoxin isoforms. The structures were generated with RNAfold (v2.3).

References:

1. Abramson, J.; Adler, J.; Dunger, J.; Evans, R.; Green, T.; Pritzel, A.; Ronneberger, O.; Willmore, L.; Ballard, A.J.; Bambrick, J.; et al. Accurate Structure Prediction of Biomolecular Interactions with AlphaFold 3. *Nature* **2024**, *630*, 493–500, doi:10.1038/s41586-024-07487-w.
